# Zn Redistribution and Volatility in ZnZrO_*x*_ Catalysts for CO_2_ Hydrogenation

**DOI:** 10.1021/acs.chemmater.3c01632

**Published:** 2023-12-11

**Authors:** Evgeniy A. Redekop, Tomas Cordero-Lanzac, Davide Salusso, Anuj Pokle, Sigurd Oien-Odegaard, Martin Fleissner Sunding, Spyros Diplas, Chiara Negri, Elisa Borfecchia, Silvia Bordiga, Unni Olsbye

**Affiliations:** † Centre for Materials Science and Nanotechnology (SMN), Department of Chemistry, University of Oslo, N-0315 Oslo, Norway; ‡ Department of Chemistry, NIS Center and INSTM Reference Center, University of Turin, Via P. Giuria 7, 10125 Turin, Italy; § Centre for Materials Science and Nanotechnology (SMN), Department of Physics, University of Oslo, N-0315 Oslo, Norway; ∥ Materials Physics Oslo, SINTEF Industry, Forskningsveien 1, NO-0373 Oslo, Norway

## Abstract

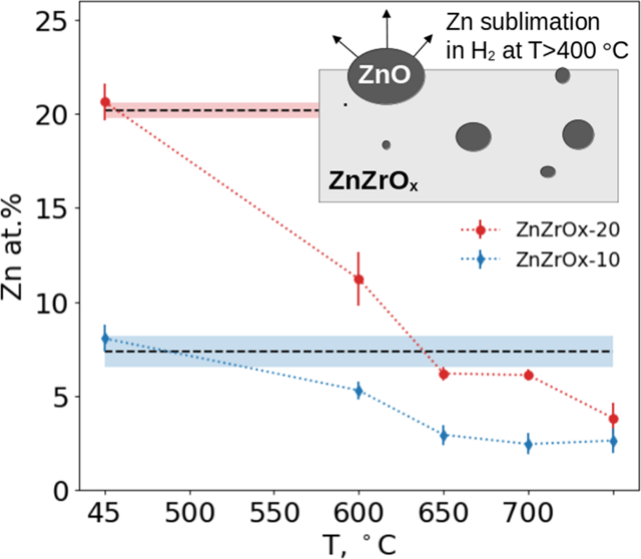

ZnO–ZrO_2_ mixed oxide (ZnZrO_*x*_) catalysts are widely studied as selective catalysts for CO_2_ hydrogenation into methanol at high-temperature conditions (300–350 °C) that are preferred for the subsequent *in situ* zeolite-catalyzed conversion of methanol into hydrocarbons in a tandem process. Zn, a key ingredient of these mixed oxide catalysts, is known to volatilize from ZnO under high-temperature conditions, but little is known about Zn mobility and volatility in mixed oxides. Here, an array of *ex situ* and *in situ* characterization techniques (scanning electron microscopy/energy dispersive X-ray spectroscopy (SEM/EDX), transmission electron microscopy (TEM), powder X-ray diffraction (PXRD), X-ray absorption spectroscopy (XAS), X-ray photoelectron spectroscopy (XPS), Infrared (IR)) was used to reveal that Zn^2+^ species are mobile between the solid solution phase with ZrO_2_ and segregated and/or embedded ZnO clusters. Upon reductive heat treatments, partially reversible ZnO cluster growth was observed above 250 °C and eventual Zn evaporation above 550 °C. Extensive Zn evaporation leads to catalyst deactivation and methanol selectivity decline in CO_2_ hydrogenation. These findings extend the fundamental knowledge of Zn-containing mixed oxide catalysts and are highly relevant for the CO_2_-to-hydrocarbon process optimization.

## Introduction

Stability of heterogeneous catalysts, along with activity and selectivity, ultimately controls their technological performance and, therefore, economic feasibility of industrial processes. Mixed oxides (MOx) containing earth-abundant elements are among the most actively investigated classes of catalytic materials because of their potential to replace more expensive and less sustainable elements, such as precious metals, in the preparation of catalysts for many industrial processes. In particular, ZrO_2_-based catalytic systems^1^ containing a secondary oxide, such as ZnO,^2,3^ CeO_2_,^4^ In_2_O_3_,^5^ or Ga_2_O_3_,^6,7^ have emerged as promising candidates for a range of important processes including CO_2_ hydrogenation to methanol and light alkane dehydrogenation. However, the structural and compositional stabilities of these systems upon exposure to the pretreatment, operating, and regeneration conditions have received relatively little attention in the literature in comparison with their activity and selectivity. During such treatments, elevated temperature and high chemical potential of gaseous species often cause drastic restructuring of catalytic materials that may lead to deactivation and loss of active components or selectivity changes. Leaching of certain catalytic components into the effluent stream is not only detrimental to the reactor performance but may also cause considerable deterioration of the downstream equipment and compromise process safety. Conversely, in some cases, treatment-induced restructuring may lead to the *in situ* emergence of more favorable surface states with enhanced catalytic performance. This motivates systematic studies of how mixed oxide catalysts restructure under the dynamic conditions that encompass not only the target catalytic reaction but also the pretreatment and regeneration.

Zn volatility is a particularly important factor to consider in the design of catalysts for the hydrogenation of CO_2_ into methanol. Sublimation of Zn species from the bulk ZnO has been known for many decades and has been employed for the industrial CVD of semiconducting ZnO films.^8^ More recently, the selective deposition of Zn sublimated from ZnO onto silanol nests of mesoporous silicas and zeolites was used to prepare highly active and selective propane dehydrogenation catalysts.^3^ In the prototypical commercial Cu/ZnO/Al_2_O_3_ methanol synthesis catalyst, partially reduced Zn in alloy with Cu gives rise to active sites for the catalytic cycle.^9,10^ It has long been known that Zn volatilizes into the gas phase from these catalysts, and controlled volatilization of Zn has even been employed to develop material recycle strategies.^11^ Bimetallic PdZn-alloyed nanoparticles provide another example of a selective methanol catalyst with considerable Zn mobility.^12,13^ In-hybrid CO_2_-to-hydrocarbon catalysts, in which PdZn nanoparticles (NPs) generate methanol to be further reacted on an acidic zeolite into hydrocarbons, Zn was shown to migrate into the zeolite framework and cause catalyst deactivation by blocking the active Bronsted acid sites. In this context, surprisingly little is known about Zn volatility in mixed ZnZrO_*x*_ oxides, which are widely investigated as potential high-temperature CO_2_ hydrogenation components for the in-hybrid oxide/zeolite (OXZEO) catalysts for the CO_2_-to-hydrocarbon conversion processes.^14^

Consensus regarding the exact structure of ZnZrO_*x*_ catalysts and the nature of the active sites is still evolving in the literature. Originally thought of as random solid solutions,^2^ coprecipitated materials were later shown by Salusso et al.^15^ to contain nanosized ZnO clusters embedded into the ZrO_2_ matrix, and it was proposed that the reaction occurs on the perimeter Zn–O–Zr sites at the ZnO–ZrO_2_ interface. More recently, Tada et al.^16^ arrived at similar conclusions for samples prepared *via* the impregnation route. Namely, a combination of multiple structural and spectroscopic techniques suggested that Zn is present in both atomically dispersed and clustered forms and that the reaction occurs in either case at the Zn–O–Zr sites. Notably, Zn was inhomogeneously dispersed with an increased concentration at the near-surface region. Feng et al.^17^ provided compelling spectro-kinetic (DRIFTS-MS) evidence and density functional theory (DFT) modeling that support the assignment of asymmetric Zn–O–Zr sites as responsible for CO_2_ hydrogenation into methanol, albeit in their case, these sites were attributed to atomically dispersed solid solution. To summarize, it is likely that the surfaces of these catalysts, depending on the preparation method, expose Zn atoms in various local environments including individual ions substituted into the ZrO_2_ matrix, Zn on the surface and at the interface of nanoclustered ZnO, and Zn within larger segregated ZnO domains. The relative prevalence of these environments under reaction conditions is an important factor that can potentially be used for practical process optimization if sufficient fundamental understanding of nanostructured ZnZrO_*x*_ catalysts is achieved.

Aside from the thermodynamically controlled Zn solubility in ZrO_2_ and the total Zn content, the kinetically controlled synthesis and pretreatment history can be used to optimize the catalytic properties. Temvuttirojn et al.^18^ have established that the temperature of the initial catalyst (oxidative) calcination significantly affects subsequent methanol selectivity under reaction conditions, which the authors attributed to variations of surface basicity. Temperature variation studies of these catalysts under reductive conditions, on the other hand, are typically limited to temperature-programmed reduction (TPR) experiments and hardly any literature report addressed the influence of the reduction temperature on the CO_2_ hydrogenation performance. TPR distinguishes several H_2_ consumption peaks ascribed to different Zn species: highly dispersed ZnO, Zn–O–Zr, or bulk ZnO. It is also known^3^ that bulk ZnO evaporates in reductive environments above 500 °C. Therefore, we hypothesize that an optimal reduction temperature can be found for ZnZrO_*x*_ materials, such that the amount of stable Zn species and associated O vacancies are maximized at the catalyst surface for improved methanol production from CO_2_. However, the dynamic restructuring among possible Zn species and Zn sublimation into the gas phase in mixed ZnZrO_*x*_ materials is not well understood.

Herein, we examine Zn redistribution and volatility within mixed oxide ZnZrO_*x*_ catalysts under various pretreatment conditions, focusing on the role of these processes in catalytic CO_2_ hydrogenation and also extending the fundamental knowledge of the material chemistry of Zn-containing mixed oxides. We employ an array of *ex situ* and *in situ* characterization techniques to evidence and quantify Zn redistribution and, eventually, loss in technologically relevant ZnZrO_*x*_ materials, which is highly dependent on the temperature and gaseous composition. The impact of material restructuring (ZnO domain growth and reversible surface segregation) and Zn volatility on the CO_2_ hydrogenation process is discussed.

## Experimental and Theoretical Methods

### Material Synthesis

In order to quantify the extent of Zn volatility and its dependency on the pretreatment conditions, ZnZrO_*x*_ materials were prepared by the coprecipitation technique of Wang et al.^2^ with nominally 0, 10, and 20 atom % of Zn, *i.e.*, Zn/(Zn + Zr). As an example, to prepare the ZnZrO_*x*_-10 sample, 1.8 g of Zn(NO_3_)_2_·6H_2_O and 24.8 g of ZrO(NO_3_)_2_·*x*H_2_O were dissolved in 150 mL of type II H_2_O in a round-bottomed flask. This was then heated to 70 °C while stirring. Twelve grams of (NH_4_)_2_CO_3_ dissolved in 50 mL of type II H_2_O was then added dropwise to the hot precursor solution. The formation of a white precipitate occurred immediately. The mixture was further stirred at 70 °C for 2 h, then cooled to ambient temperature, isolated by centrifugation, and washed twice with type II H_2_O. Wet samples were then oven-dried at 110 °C and calcined at 600 °C for 3 h in air. A nominal Zn content in these parent samples is used in sample names throughout the manuscript (ZnZrO_*x*_-NN). According to many reports,^2,19−21^ methanol yield increases with the increasing bulk concentration of Zn only up to 10–15 atom %, which is believed to be limited by Zn solubility in the ZrO_2_ matrix before a less active and selective ZnO phase segregates from the solution. Correspondingly, the sample with 10 atom % Zn was the main focus of this work, representing the compositionally optimal CO_2_ hydrogenation catalyst for the tandem OXZEO process.^19^ The 20 atom % Zn sample was additionally used to facilitate *in situ* powder X-ray diffraction (PXRD) measurements, which are poorly sensitive at lower Zn concentrations. Most types of measurements were performed on both 10 and 20 atom % samples to ensure the generality of conclusions. A control ZrO_2_ sample without Zn was synthesized by following the same protocol.

### Pretreatments and Catalytic Testing

First, samples (sieve fraction 250 < *d*_p_ < 420 μm) were pretreated under a 25% H_2_ (in Ar) continuous flow in an atmospheric-pressure tubular quartz reactor using a high-temperature ceramic oven. Three different temperatures were selected to elucidate the influence of pretreatment on catalyst performance: 400, 550, and 700 °C. A temperature ramp of 5 °C min^–1^ was used, and the final temperature was kept constant for 4 h before cooling the reactor and extracting the sample. All lines before and after the reactor were heated to 150 °C. The maximum temperature of pretreatment is indicated at the end of the sample name when necessary, *e.g.*, ZnZrO_*x*_-10–550 denotes a sample with 10% atom Zn was pretreated in H_2_ at 550 °C.

Pretreated samples were loaded in a high-pressure test reactor (PID Eng and Tech) for catalytic activity testing. The equipment consisted of a silica-lined packed bed stainless steel reactor (inner diameter 9 mm) heated by a cylindrical ceramic oven and provided with a downstream pressure regulator able to maintain the system up to 60 bar. Temperature was measured and controlled by a K-type thermocouple placed inside the catalytic bed. The reactor was located inside a hot box at 160 °C, where the gases were premixed and heated and downstream gases were maintained hot. Reaction products were monitored using a gas chromatograph (Agilent 8890 GC) connected in line with the reactor. The line between the reactor and the GC was heated at 150 °C to avoid product condensation. The GC was provided with three columns (CP-Sil 8 CB, GS-GasPro, CP Molesieve 5A), several detectors (two FIDs and a TCD), and a PloyARC microreactor for a reliable quantification of CO_2_ and CO. Prior to catalytic testing, the reactor temperature was increased up to 400 °C with a heating rate of 5 °C min^–1^ under a similar 25% H_2_ (in Ar) flow for 4 h, after which the temperature was decreased to the reaction temperature and pressure was increased. In a typical experiment, CO_2_ hydrogenation runs were carried out at 350 °C, 30 bar, and GSHV values of 12,000–48,000 cm^3^ h^–1^*g*_ZnZrO*_x_*_^–1^. Differential reaction conditions were aimed to avoid the thermodynamic restrictions of CO_2_ hydrogenation to methanol and, therefore, compare samples in the kinetic regime. Each run was repeated a minimum of 5 times, and the initial tested conditions were repeated at the end of the experiment to ensure the reproducibility of the results. Values reported herein are the average of all experiments with analytical errors lower than 2%. CO_2_ conversion, product selectivity, and space time yield (STY) were used to monitor the reaction, and their definitions can be found in the Supporting Information.

In a separate series of experiments, similar high-temperature H_2_ pretreatments of parent ZnZrO_*x*_-10 and ZnZrO_*x*_-20 samples were performed at 400, 550, 600, 650, and 700 °C. In each case, the sample was placed on top of a thin (ca. 2 mm) quartz frit that physically separated the sample from a catchment layer of pure ZrO_2_ (same sieve fraction) positioned immediately downstream from the frit (see Figure S1). The ZrO_2_ layer, in turn, was supported by another quartz frit. After the high-temperature pretreatment (4 h in a H_2_/He flow at the final target temperature), a quartz reactor was cut at the position of the interlayer frit, and the sample and catchment layers were carefully separated and removed for *ex situ* analysis.

### Material Characterization

#### Energy-Dispersive X-ray (EDX)

Energy-dispersive X-ray spectroscopy was performed using a Hitachi SU8230 scanning electron microscope (SEM) operated at a 20 kV acceleration voltage, a 1 k magnification, and a 30 μA current. Both Zn and Zr were quantified at their respective L-edges (see the example in Figure S2).

#### X-ray Photoelectron Spectroscopy/Auger Electron Spectroscopy (XPS/AES)

The spectra were collected with a Kratos Axis Ultra^DLD^ instrument by using monochromatic AlKα radiation (1486.6 eV) at 15 kV and 10 mA emission. A pass energy of 20 eV was used to collect all spectra, and low-energy electrons were used for charge compensation. For each sample, the Zr 3d_5/2_ peak position was used as an internal energy reference, which fell within the binding energy (BE) range of around 180–181 eV consistent with Zr^2+^. Prior to XPS data collection in “near-*in situ*” experiments, samples were pretreated inside a chemical treatment cell interfaced with the XPS analysis chamber *via* air-free sample transfer. Samples were exposed to a 3.5% H_2_ (in Ar) flow at the total pressure of 1 bar, while the temperature was ramped to 650 °C at 10 °C min^–1^, followed by a 15 min isothermal soak. Then, the sample was allowed to cool to room temperature under a H_2_/Ar flow, the chemical treatment cell was evacuated, and the sample was transferred to the analysis chamber in vacuum.

#### *Ex Situ* X-ray Absorption Spectra (XAS)

*Ex situ* X-ray absorption spectra were collected in a transmission mode on 13 mm^2^ pellets with a Si(111) double-crystal monochromator at the BM31 beamline of the European Synchrotron Radiation Facility (ESRF). Zn K-edge spectra were measured in the 9.5–10.2 keV energy range with a 0.5 eV step size. Spectra of reference ZnO (Fluka, > 99%) were measured with the same parameters. Zr K-edge spectra were measured in the 17.9–18.7 keV energy range with a 0.5 eV step size. Both edges were recorded with a 0.1 s/point exposure time for a total of ≈2.5 min/scan. Six scans/samples/edges were collected and averaged after energy alignment, background subtraction, and edge-jump normalization conducted with the ATHENA software from the Demeter Package.^22^ The ratio between Zn K- and Hf L_3_-edge steps was calculated as
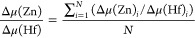
where *N* is the number of collected spectra. The edge step was obtained from the difference between μ(*E*) extracted from pre-edge and postedge lines (see Figure S6a,b). The values reported in Figure S6c refer to the average edge step ratios of six scans, and the reported error was evaluated as their standard deviation. The calibration line was obtained with a linear fit (*y* = *mx* + *q*) of the three samples. The error on the Zn at % calculated with the calibration line was evaluated as
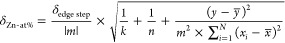
where m is the slope of the calibration line, *k* is the number of replica (*i.e.*, 6), and *n* is the number of calibrants (*i.e.*, 3). *y* is the average of the sample edge step, and *y̅* is the average of the calibrant signals. *x_i_* is the *i*th evaluated Zn atom %, while *x̅* is the average value of the Zn content. The reported extended X-ray absorption fine structure (EXAFS) fits were conducted with the ARTEMIS software from the Demeter Package.^22^

#### *Ex Situ* and *In Situ* Powder X-ray Diffraction

*Ex situ* and *in situ* powder X-ray diffraction data were collected with a monochromatic beam (λ ≈ 0.274 Å) at the same beamline. Catalysts were finely ground and packed in a quartz capillary (Ø = 1 mm). For *in situ* experiments, the capillary was connected to a gas flow system, sending 25 sccm of a 1:1 H_2_/He stream, using a heat blower to control the temperature. Debye–Scherrer rings were collected by a CCD camera with a 100 ms/scan exposure time in the 0–25° 2θ range. Five scans were collected, and one-dimensional (1D) PXRD patterns were obtained after integration of the diffracted rings. The final 1D PXRD resulted from an average of 5 scans. Lattice parameters were refined for each pattern using the Rietveld refinement method implemented in FullProf software.^23^ Refinement χ^2^ values for each pattern and refined curves for data collected at RT and 650 °C are reported in Figure S3.

#### Scanning Transmission Electron Microscopy (STEM)

Scanning transmission electron microscopy images and energy dispersive spectroscopy (EDS) elemental maps were collected using a Thermo Fisher Scientific Titan G2 60–300 kV microscope equipped with a DCOR Cs probe-corrector and Super-X EDS detectors. Experiments were performed at a 300 kV accelerating voltage using a high-angle annular dark-field (HAADF) detector. STEM samples were prepared by sonicating ZnZrO_*x*_ powders in ethanol and drop-casting the resulting suspension onto the carbon-coated TEM nickel grids.

## Results and Discussion

### Volatilization of Zn during Steady-State CO_2_ Hydrogenation at 350 °C

**Figure 1:**
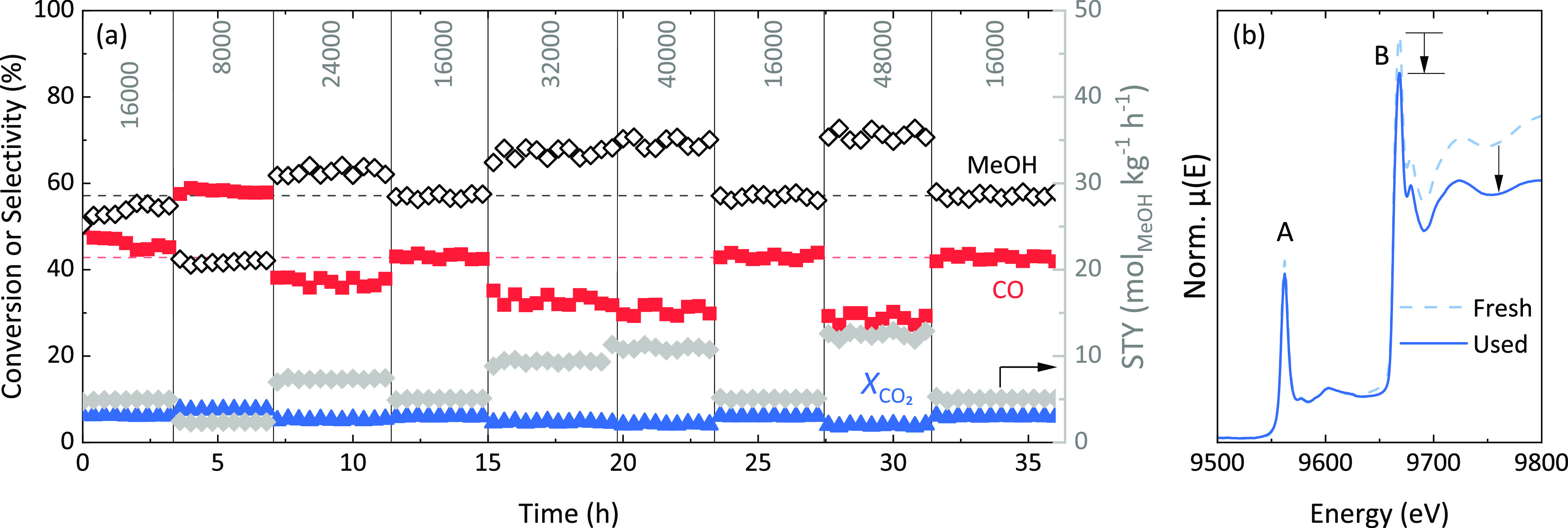
Zn loss from ZnZrO_*x*_-10–400 catalyst during CO_2_ hydrogenation at 350 °C and 30 bar (1:3 ratio CO_2_/H_2_): (a) CO_2_ conversion (X_CO2_), CO and MeOH selectivities and methanol STY as functions of time on stream at variable space velocities (in cm^3^ h^–1^ g_ZnZrO*_x_*_^–1^), (b) *ex situ* Zn K-edge XAS spectra of fresh and used catalysts, before and after the experiment in panel (a). Labeled spectral features A and B correspond to the L_3_-edge of Hf and the K-edge of Zn, respectively. Spectra are normalized to the Hf L_3_-edge. The arrows highlight the decrease in the normalized Zn signal after the reaction.

Before their use in CO_2_ hydrogenation, ZnZrO_*x*_ catalysts are typically activated in a flow of H_2_ at 400 °C and ambient pressure, presumably to remove surface contamination and create O vacancies (O_v_), which are believed to be required for the reaction.^19^ This activation procedure is followed by the exposure of catalysts to a mixture of H_2_ and CO_2_ (3:1 ratio) at 350 °C and moderate pressure (10–30 bar) to produce methanol. The starting point of this work was a curious observation made by comparing Zn K-edge XAS spectra of an activated ZnZrO_*x*_ catalyst with 10 atom % Zn (ZnZrO_*x*_-10–400) taken before and after its prolonged (>35 h) exposure to typical CO_2_ hydrogenation conditions (*i.e.*, 350 °C, 30 bar). CO_2_ conversion as well as methanol and CO selectivities during this experiment behaved in a predictable manner (Figure 1a), in accordance with the periodically altered space velocity. Between each new reaction segment, the space velocity was returned to 16,000 cm^3^ h^–1^ g_ZnZrO*_x_*_^–1^ for several hours in order to reference the catalyst performance to a reproducible standard state. During the first segment with these standard conditions, the methanol selectivity slightly increased at the expense of CO but it remained constant during all subsequent reference segments, suggesting that the catalyst performance was robustly returned to the same state after each excursion in space velocity and for many hours on stream. However, it was noticed that measurable changes in XAS spectra have occurred during this time when comparing spectra for the fresh and used catalysts (Figure 1b). Here, two pronounced absorption edges and white-line peaks can be distinguished, corresponding to the (B) Zn K-edge and (A) Hf L_3_-edge, respectively. The presence of Hf in the catalyst is expected, as Hf commonly accompanies Zr in commercially available synthesis precursors due to their natural collocation in Zr ores.^24^ When normalized to Hf edge jump, these spectra clearly reveal the decrease of Zn white-line and edge jump, which coupled with the high Hf stability in the ZrO_2_ lattice suggests a gradual loss of Zn under reaction conditions.

These preliminary observations raise a number of questions. How does the history of pretreatment and reaction conditions, *i.e.*, temperature and the reduction/oxidation potential of the gas, control the migration and loss of Zn from ZnZrO_*x*_ mixed oxides? Why does the loss of Zn from the catalyst bulk, at least in moderate amounts, not affect its performance in the hydrogenation of CO_2_ to methanol? To answer these questions, we undertook a systematic study of Zn redistribution and volatility as a function of treatment conditions. In order to understand the material behavior, we subjected the ZnZrO_*x*_ not only to relevant conditions of temperature and gas composition for the process, but also to harsher conditions, aiming to analyze the stability and nature of Zn species in the mixed oxide.

### “Near-*In Situ*” XPS Evidence of Gaseous Zn Transfer

**Figure 2:**
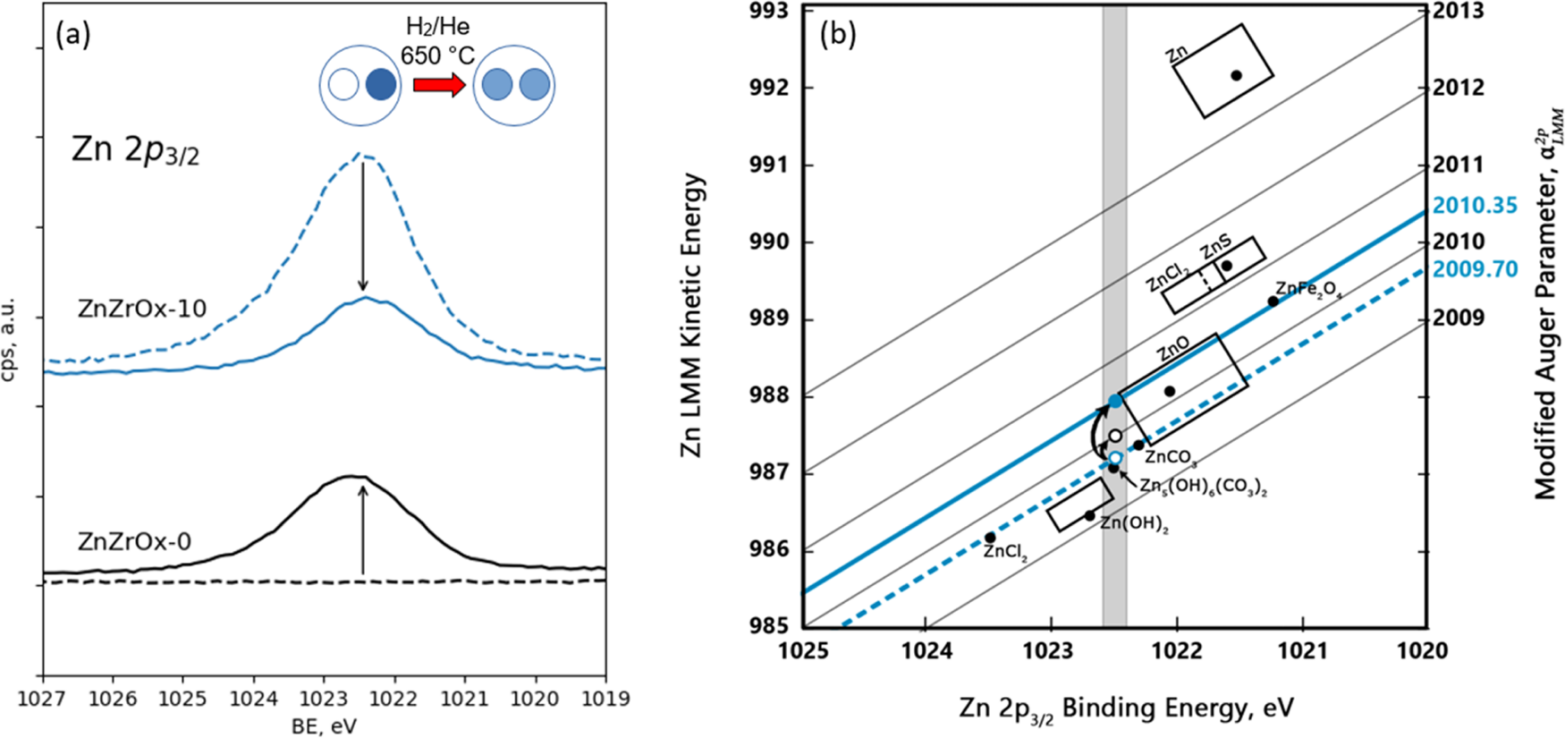
“Near-*in-situ*” XPS characterization of Zn transfer during sample reduction. (a) Zn 2p_3/2_ XPS for ZnZrO_*x*_-0 and ZnZrO_*x*_-10 samples before and after reduction in 5% H_2_/He (1 bar total pressure) at 650 °C. The inset schematically depicts the color change in two adjacent sample wells, with the color depth corresponding to the Zn concentration. (b) Modified Auger parameter map. The original state (open blue circle) with an α′_LMM_^2p^ value of 2009.70 (dashed blue line) is shown to transform into the residual state (full blue circle) with an α value of 2010.35 (full blue line) after reduction and Zn sublimation. Zn from the downstream ZrO_2_ catchment layer (open black circle) exhibits an α value of 2010. All three states are characterized by a range of binding energies within 1022.4–1022.6 eV, as indicated by the vertical gray-shaded area. Full black circles represent the α value of reference compounds (data adopted from Dake et al.^25^), with rectangles reflecting the spread of the literature values.

First, “near-*in situ*” XPS measurements were performed in order to directly evidence the gas-phase-mediated transport of Zn and to gain insight into its chemical state before and after high-temperature reductive treatment. ZnZrO_*x*_-10 and ZnZrO_*x*_-0 (pure ZrO_2_) samples were placed into two individual, spatially separated wells in the same quartz holder and subjected to a flow of 5% H_2_ at 650 °C and a total pressure of 1 bar. A significantly higher reduction temperature in this experiment was used in order to accelerate possible Zn evaporation in comparison with the aforementioned experiment under standard conditions (Figure 1). Zn 2p_3/2_ spectra before and after treatment are shown in Figure 2a. The appearance of a prominent Zn 2p peak in the spectrum of the originally Zn-free ZrO_2_ after the treatment and the simultaneous decrease in the Zn 2p intensity in the spectrum of the ZnZrO_*x*_-10 sample clearly corroborate gas-phase Zn transfer between the samples. We note that multiple spots on the sample surface were examined to exclude erroneous results due to the physical transfer of powder particles between the wells.

The main component of the core Zn 2p_3/2_ peak in all Zn-containing samples had a binding energy of 1022.4–1022.6 eV (see Figure S4), which is consistent with the Zn^2+^ oxidation state.^25^ No systematic changes in the chemical shift of this peak could be identified after exposure to reducing environment and intersample Zn transfer. The Zn 2p core level is generally insensitive to variations of the Zn chemical state, aside from the formal oxidation state, and its in-depth interpretation is further aggravated by referencing the binding energy scale to the Zr 3d level, which may be affected by the reduction process.^26^ In the absence of a reliable internal reference, the Zn chemical state was instead inferred from the modified Auger parameter defined as
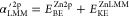
where Zn 2p BE, eV and Zn LMM KE, eV are the binding energy of the 2p_3/2_ core electron and the kinetic energy of the LM_45_M_45_ secondary Auger electron, respectively. The Auger parameter is plotted in Figure 2b along known reference compounds.^25^ Zn species on the surface of the ZnZrO_*x*_-10 sample before pretreatment exhibited the Auger parameter within 2009.7–2009.8 eV, which is consistent with the ZnCO_3_ reference and suggests that surfaces of ZnZrO_*x*_ catalysts are covered with carbonates that are formed during air exposure. After reduction, the Auger parameter shifted to 2010.35–2010.4 eV, located at the boundary of literature-reported values for ZnO. This shift is consistent with the decomposition of carbonates and partial reduction of Zn, *e.g.*, formation of O vacancies. Complementary *in situ* DRIFTS IR data recorded during TPR also confirm the presence of surface Zn carbonates^27,28^ (bands at 1200–1800 cm^–1^) that decompose upon heating in H_2_ (see Figure S5). Interestingly, gaseous Zn species that landed on a pure ZrO_2_ catchment layer exhibited a somewhat lower Auger parameter of 2010.0 eV, suggesting that even if Zn(0) species cannot be excluded as the gas-phase transport intermediate, it immediately oxidizes upon deposition on the ZrO_2_ substrate and forms ZnO domains. Similar observations were confirmed by experiments with the ZnZrO_*x*_-20 sample (see Figure S4).

### SEM/EDX Quantification of Zn Transfer

**Figure 3:**
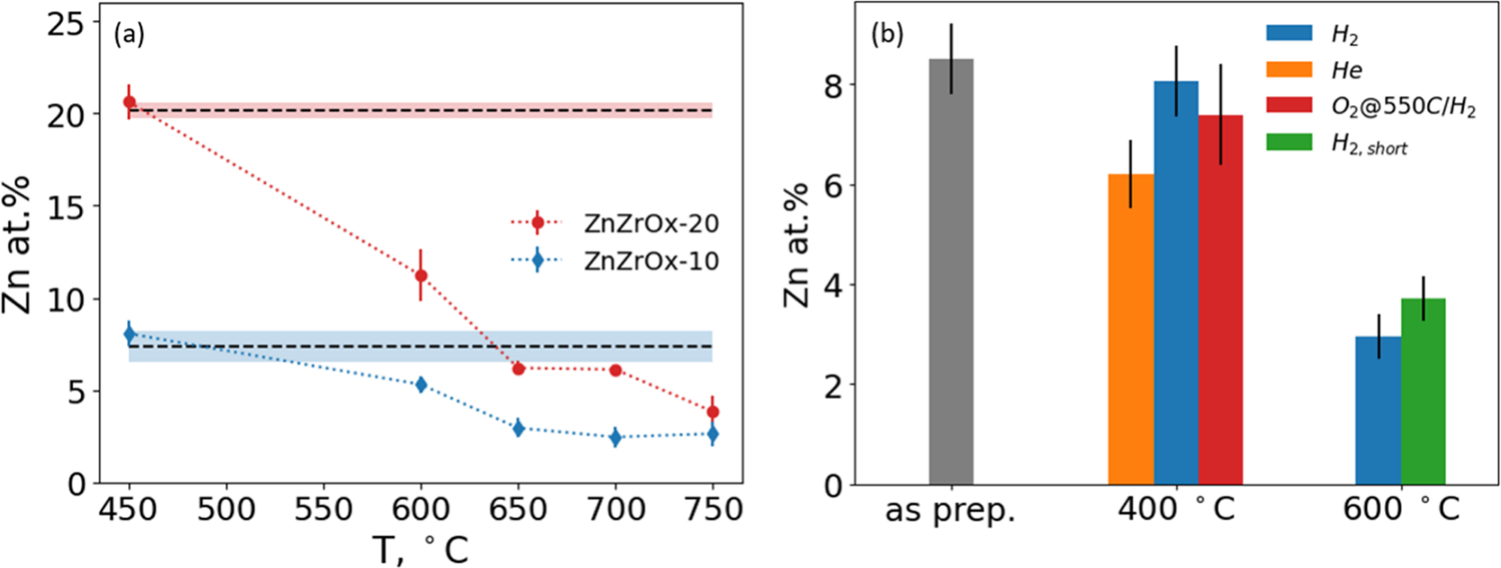
Sublimation experiments and residual Zn concentration in catalyst samples quantified by EDX. (a) Residual Zn concentration *vs* H_2_/He pretreatment temperature. The initial Zn concentration in the two as-prepared samples is indicated by the horizontal straight lines (dashed, mean values; shaded area, one standard deviation), and post-treatment concentration is shown as points with error bars. (b) Residual Zn concentration in the ZnZrO_*x*_-10 sample as a function of gas environment, temperature, and the treatment duration.

Next, having determined that ambient-pressure high-temperature pretreatment in H_2_ can lead to Zn evaporation, the temperature dependency of Zn loss from ZnZrO_*x*_ catalysts was quantified using SEM/EDX (see the example analysis in Figure S2). Figure 3a compares the evolution of the residual Zn content as a function of H_2_ pretreatment temperature for ZnZrO_*x*_-10 and ZnZrO_*x*_-20 samples. Zn concentration does not change significantly in either sample after their exposure to H_2_ at 400 °C. However, it rapidly decreases upon further increase in the pretreatment temperature; more than half of Zn is lost from both samples within the 550–600 °C range, above which the residual Zn concentration converges around 3 atom %. for both samples. The decrease in Zn concentration with increasing pretreatment temperature can be unequivocally attributed to the loss of Zn into the gas phase rather than its migration into the bulk of the material, based on the relatively high penetration depth of EDX analysis and direct observations of Zn deposition on the initially Zn-free substrates downstream from the source material (*vide infra*).

Additional experiments demonstrated that the gas composition, in addition to temperature, plays a crucial role in controlling Zn sublimation but not the duration of treatment. In Figure 3b, the residual Zn concentration is compared to its initial value in the ZnZrO_*x*_-10 sample after several pretreatment protocols. As already seen in Figure 3a, the treatment at 400 °C in H_2_ did not cause a significant Zn loss in comparison with the treatment at 600 °C in H_2_. In another experiment mimicking a realistic catalyst regeneration procedure, *i.e.*, periodic coke burnoff during the OXZEO process, the material was first exposed to O_2_ at 550 °C, followed by H_2_ at 400 °C. The treatment in O_2_ at 550 °C, however, did not decrease the Zn concentration, suggesting that reducing environment is critical for Zn sublimation above 400 °C. Curiously, treatment in helium even at 400 °C resulted in a slight but measurable Zn loss. The role of the exposure duration at high temperature in H_2_ on the rate of Zn loss was investigated by comparing the residual Zn content after a 4 h treatment at 600 °C and after a brief (<5 min) exposure at that temperature with subsequent rapid quenching to room temperature. Approximately the same amount of Zn was lost during the rapid temperature excursion and a prolonged treatment, which suggests that Zn sublimation occurs on a relatively short time scale at these conditions.

### *Ex Situ* XAS Characterization of Zn Species

**Figure 4:**
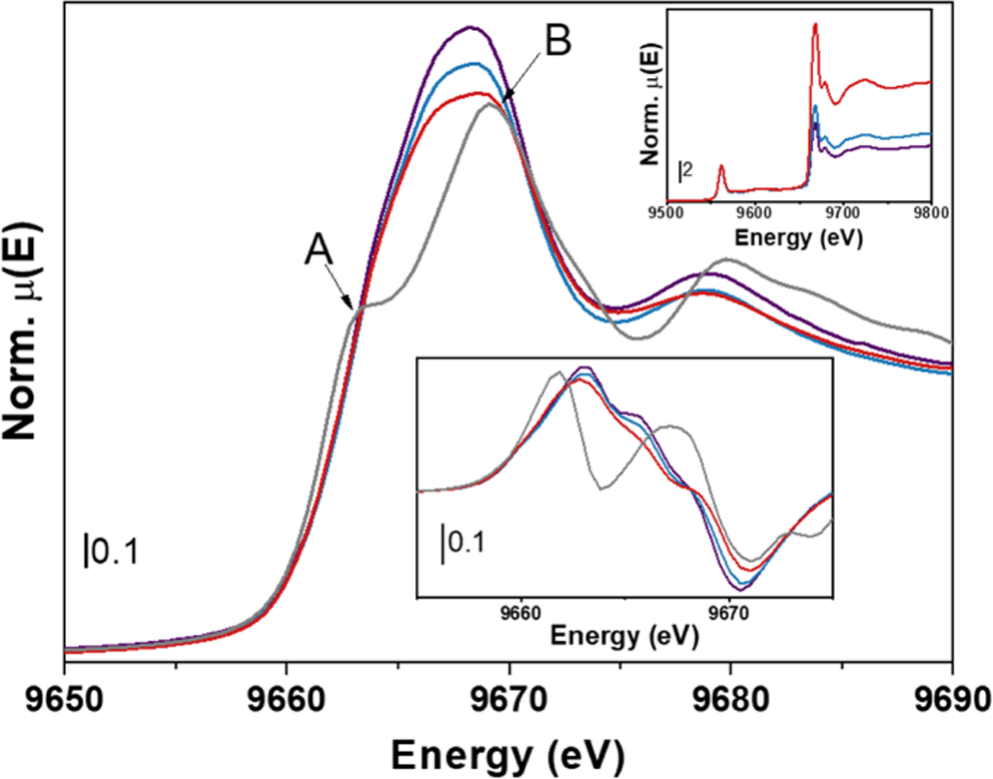
Zn K-edge (main panel) and Hf L_3_-edge (top inset) normalized XANES spectra for ZnZrO_*x*_-5 (purple line), ZnZrO_*x*_-10 (blue line), ZnZrO_*x*_-20 (red line), and ZnO (gray line). The first derivative spectra for the same samples are reported in the bottom inset.

Zn K-edge XAS is known to be sensitive to the state of Zn in ZnZrO_*x*_ samples, as already mentioned in Figure 1b. *Ex situ* XAS spectra at Zn K-edge are reported in Figure 4 for the parent (*i.e.*, calcined but not reduced) samples, with Zn concentration increasing from ZnZrO_*x*_-5 (from a previous work^15,19^) to ZnZrO_*x*_-10 and ZnZrO_*x*_-20. Zn K-edge of a reference h-ZnO material is also shown for comparison. According to Lee et al.,^29^ the two main contributions in these spectra are related to 1s → 4p_π_ (label A) and 1s → 4p_σ_ (label B) transitions associated with h-ZnO axial and in-plane hybridizations, respectively. In Zn-doped ZrO_2_ catalysts, Zn has been shown to form ZnO nanoclusters rather than a solid solution.^15^ Thus, Zn K-edge X-ray absorption near edge spectroscopy (XANES) spectra in Figure 4 indicate that ZnO clusters are prevalently formed along the in-plane direction of the hexagonal lattice. As the Zn concentration in the catalysts increases, a decrease in white-line intensity is observed, suggesting a lower availability of unoccupied states. This trend can be understood by considering the dependency of unoccupied states on the ZnO cluster size. Following Lee et al.,^29^ ZnO particles of smaller diameters are expected to have larger surface-to-bulk atomic ratios with enhanced unoccupied surface states near conduction band minimum. Less unoccupied states, then, can be associated with lower surface/bulk ratios, consistent with larger ZnO clusters in ZnZrO_*x*_ catalysts with higher Zn concentration.

The spectral range at slightly lower energies from the Zn K-edge (see the top right inset in Figure 4) reveals the presence of the Hf L_3_-edge. As explained before, Hf is a natural contaminant in ZrO_2_, which can be exploited as an internal standard for direct quantification of Zn atom %. While the relative Hf abundance with respect to Zr is the same in each catalyst in the series, since they share the same Zr precursor salt, the Δμ(Zn)/Δμ(Hf) ratio reflects changes with Zn loadings. Indeed, it can be observed that Zn edge jump normalized by the Hf L_3_-edge becomes more pronounced as the Zn concentration increases. The Δμ(Zn)/Δμ(Hf) ratio was used to build a calibration line with Zn atom % (see Figure S6).

**Figure 5:**
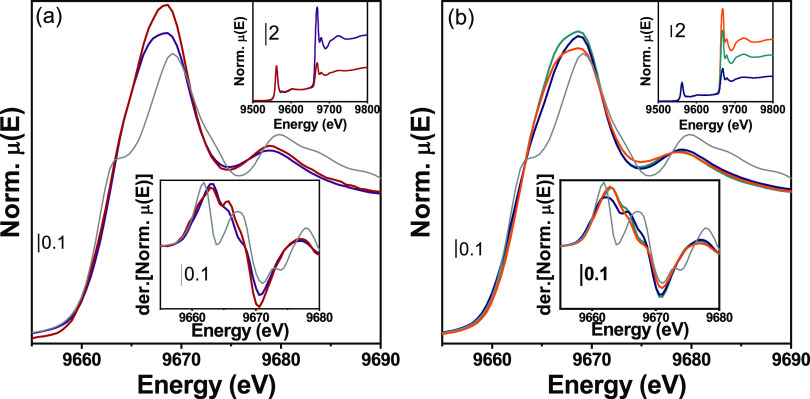
Zn K-edge XANES spectra of (a) ZnZrO_*x*_-10 (dark red line), ZnZrO*_x_*-10–700 (purple line), and (b) ZnZrO*_x_*-20 as-prepared (orange line), ZnZrO*_x_*-20–550 (light blue line), and ZnZrO*_x_*-20–700 (dark blue). h-ZnO reference spectrum is reported in gray. XANES spectra normalized at Hf L3-edge are shown in the top inset. XANES first derivative spectra are reported in the bottom insets in both panels.

The aforementioned trends in Zn K-edge spectra of as-prepared materials with known (bulk) Zn concentration become useful when considering spectra with unknown Zn concentration, namely, ZnZrO_*x*_-10 and ZnZrO_*x*_-20 after thermal treatments in H_2_ at 550 and 700 °C (Figure 5). The Δμ(Zn)/Δμ(Hf) (insets in Figure 5 and table in Figure S6c) decreases as the reduction treatment temperature increases, indicating a decrease in Zn concentration consistent with EDX data in Figure 3. Indeed, using the Hf signal as a reference, we estimate that the Zn content decreased to 13.78 ± 0.02 and 4 ± 3 atom % in ZnZrO*_x_*-20–550 and ZnZrO*_x_*-20–700 samples, respectively, while it became unmeasurable (0 ± 4 atom %) in ZnZrO*_x_*-10–700. The high uncertainty on the latter value has to be related to the low Δμ(Zn)/Δμ(Hf) (1.53 ± 0.04), which falls far from the calibrant average value. Along with the decrease of the Zn concentration, individual components comprising Zn K-edge 1s → 4p_σ/π_ transitions become more discernible after H_2_ treatment. In particular, spectral component B in ZnZrO_*x*_-20–550 increases (Figure 5b), while both A and B components become more distinct and less intense in ZnZrO_*x*_-20–700 (Figure 5b) and ZnZrO_*x*_-10–700 (Figure 5a). These observations suggest that the ZnO cluster dimension first decreases with the loss of Zn concentration, while it subsequently increases at higher treatment temperatures. The same trend can be qualitatively observed from the EXAFS region (Figure S7). Even though an EXAFS fit including the first and second shells was not achievable as discussed in the Supporting Information (SI), the EXAFS and its FT magnitude and imaginary components presented more pronounced ZnO Zn–Zn scattering path fingerprints (Figure S7) for samples treated at 700 °C, suggesting an increase of this path contribution.

Zr K-edge spectra shown in Figure S8 provide complementary information on the local structure of the ZrO_2_ matrix and also indirect clues on ZnO clusters. Zr XANES is characterized by a pre-edge peak related to the 1s → 4d transition (labeled with A in Figure S8) and a structured white-line peak, eventually split into two components (B and B′ in Figure S8). Both features, *i.e.*, pre-edge component A and the B/B′ splitting, are indicative of the tetragonal ZrO_2_ polymorph. Component A is associated with the noncentrosymmetricity of the absorber, directly distinguishing the tetragonal polymorph from the cubic one.^30^ The B/B′ splitting is another typical fingerprint of the tetragonal polymorph that would not have been observed in the monoclinic one. Moreover, the Zr EXAFS region is very informative on the ZnO–ZrO_2_ interface. The Zn–Zr scattering path was observed in the Zr EXAFS second shell region since the antiphase configuration between Zr–Zr_ZrO_2__ and Zr–Zn_int_ paths, involving Zn atoms at the cluster interface, caused a decrease in its intensity. Indeed, the FT-EXAFS fit of the reported spectra (Figure S10 and Table S2) indicated that only thermal treatment at 700 °C clearly affected Zr–Zn CN. This is clearly visible in ZnZrO-20 where the Zn initial content is sufficiently high to allow its reasonable estimation (Table S2). As rationalized hereafter by catalytic tests, the evaluated Zr–Zn CN indicates as after treatment at 550 °C the Zn content decreases, however, without affecting the Zr–Zn interface. Contrarily after treatment at 700 °C, the Zr–Zn contribution is negligible, highlighting as Zn restructuring strongly affected the Zr–Zn interface.

### *In Situ* PXRD during Zn Redistribution and Volatility

Additional insights into the process of Zn redistribution and volatilization were gained from powder X-ray diffraction, which is sensitive to the (bulk) ZrO_2_ polymorphism and lattice spacing. *Ex situ* PXRD data collected on the same samples before and after reductive pretreatment (see Figures S11 and S12) showed that Zn initially stabilized the tetragonal phase of ZrO_2_, but monoclinic reflections intensified after H_2_ treatment, suggesting a slight decrease in the fraction of the ZrO_2_ tetragonal polymorph in favor of the monoclinic one. Moreover, H_2_ reduction caused a shift of the t-ZrO_2_ reflections to lower angles, which is indicative of an increase of the average unit cell volume caused by the migration of Zn out of the ZrO_2_ matrix.^15^

**Figure 6:**
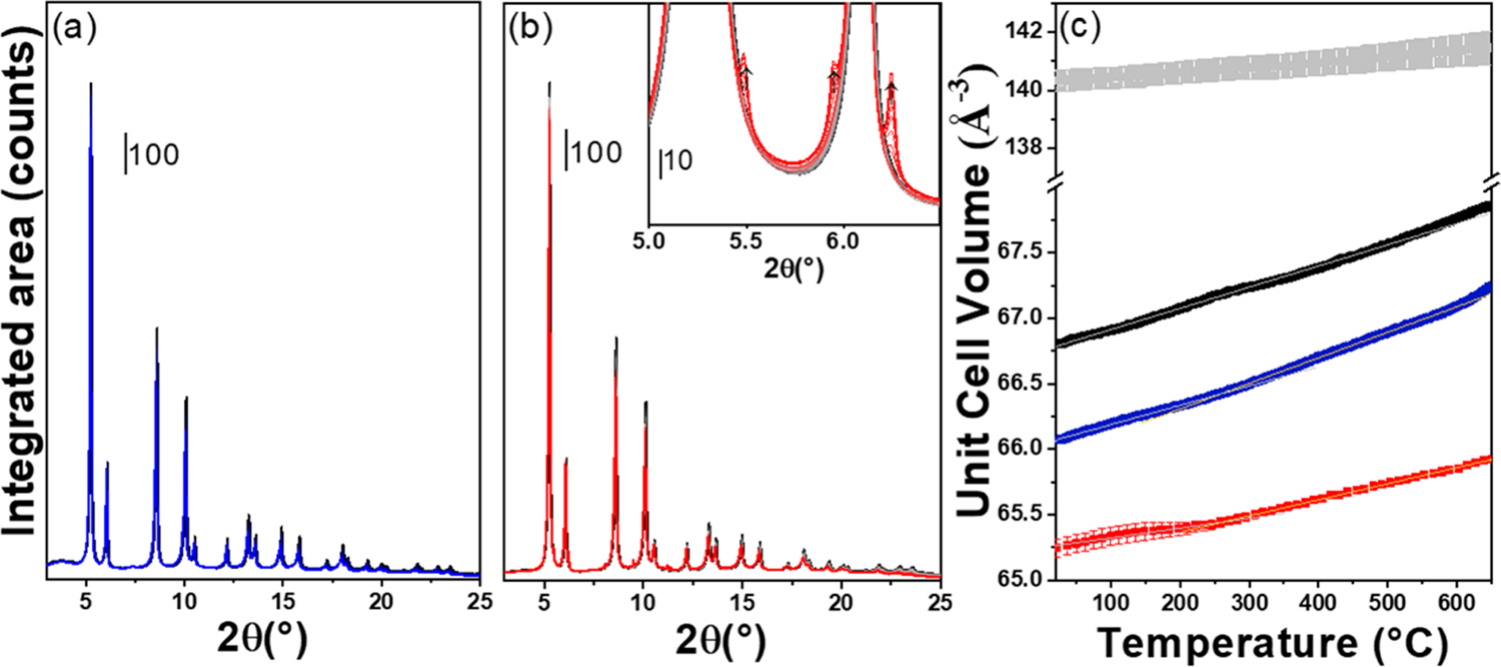
PXRD pattern collected for (a) ZrZnO*_x_*-10 and (b) ZrZnO*_x_*-20 during H_2_-TPR experiment. The temperature increases from RT (black line) to 650 °C (colored line) under a 50% H_2_ (in He) flow. The detail of ZnO reflections, indicated with arrows, is reported in the inset in part (b). (c) Unit cell volume thermal evolution for reference t-ZrO_2_ (black line), m-ZrO_2_ (gray line), ZnZrO_*x*_-10 (blue line), and ZnZrO_*x*_-20 (red line). Linearity regions are shown with colored lines.

In order to understand the process of Zn loss in more detail, *in situ* PXRD patterns were collected for pure ZrO_2_, ZnZrO_*x*_-10, and ZnZrO_*x*_-20 catalysts, while heating samples from RT to 650 °C under a H_2_/He stream. RT-PXRD patterns indicate that the presence of Zn stabilized the tetragonal polymorph in ZnZrO_*x*_-10 and ZnZrO_*x*_-20 catalysts (Figure 6a,b), decreasing its unit cell volume in comparison with the pure t-ZrO_2_ reference (Figure 6c). Indeed, in the latter, the absence of Zn is accompanied by the coexistence of monoclinic and tetragonal phases (32:68 wt %; see Figure S13), which ratio did not significantly change by increasing temperature. However, a clear difference was observed in the unit cell volume evolution of the three samples while changing temperature. As reported in Figure 6c, the unit cell volume of pure ZrO_2_ increases linearly until ca. 500 °C with a constant slope reflecting thermal expansion. For the ZnZrO_*x*_-10 and ZnZrO_*x*_-20 catalysts, on the other hand, the unit cell volume variation presented a slope change in the 200–250 °C range highlighted from its first derivative (Figure S14). Indeed, the volume expansion is better described by considering two linear regions rather than one, as confirmed by the F test reported in Figure S14. The first region (gray line Figure 6c) can be associated with unit cell thermal expansion, but around 250 °C, the slope becomes steeper (yellow lines, Figure 6c). Zn migration within the material, *e.g.*, its egression from the ZrO_2_ framework, and its subsequent loss into the gas phase could account for the increased slope of unit cell expansion upon heating since R(Zn^2+^) < R(Zr^4+^) according to Shannon.^31^ Moreover, at *T* > 600 °C, h-ZnO reflections started to be observed for the ZnZrO_*x*_-20 catalyst (Figure 6b, inset), confirming, as shown by XAS, that the Zn loss is accompanied by an increase in the average dimension of ZnO clusters, in which the residual Zn is organized. To summarize, *in situ* data suggest that ZnO cluster aggregation takes place already at 250 °C and proceeds further as temperature increases, until the characteristic h-ZnO reflections become visible by PXRD. Interestingly, h-ZnO reflections were not observed in *ex situ* diffractograms collected at RT after high-temperature treatments (see Figures S11 and S12). In the ZrZnO*_x_*-20 case, the intensity of h-ZnO reflections decreased during cooling under H_2_ (see Figure S15a), suggesting that the dimensions of ZnO domains spontaneously decrease at lower temperatures. Moreover, careful analysis of the lattice parameter during cooling (Figure S15b,c) demonstrates the presence of two lattice contraction regions: (I) a high-temperature one (650 °C > *T* > 450 °C) characterized by a Zn-poor ZrO_2_ lattice and (II) a low-temperature one (*T* < 450 °C) with higher slope, hence identifying the enrichment of ZrO_2_ with Zn. These outcomes strengthen the conclusion that ZnO clusters break up into smaller domains upon cooling and then partially dissolve into the ZrO_2_ lattice.

### *Ex Situ* TEM at Different Stages of Zn Loss

**Figure 7:**
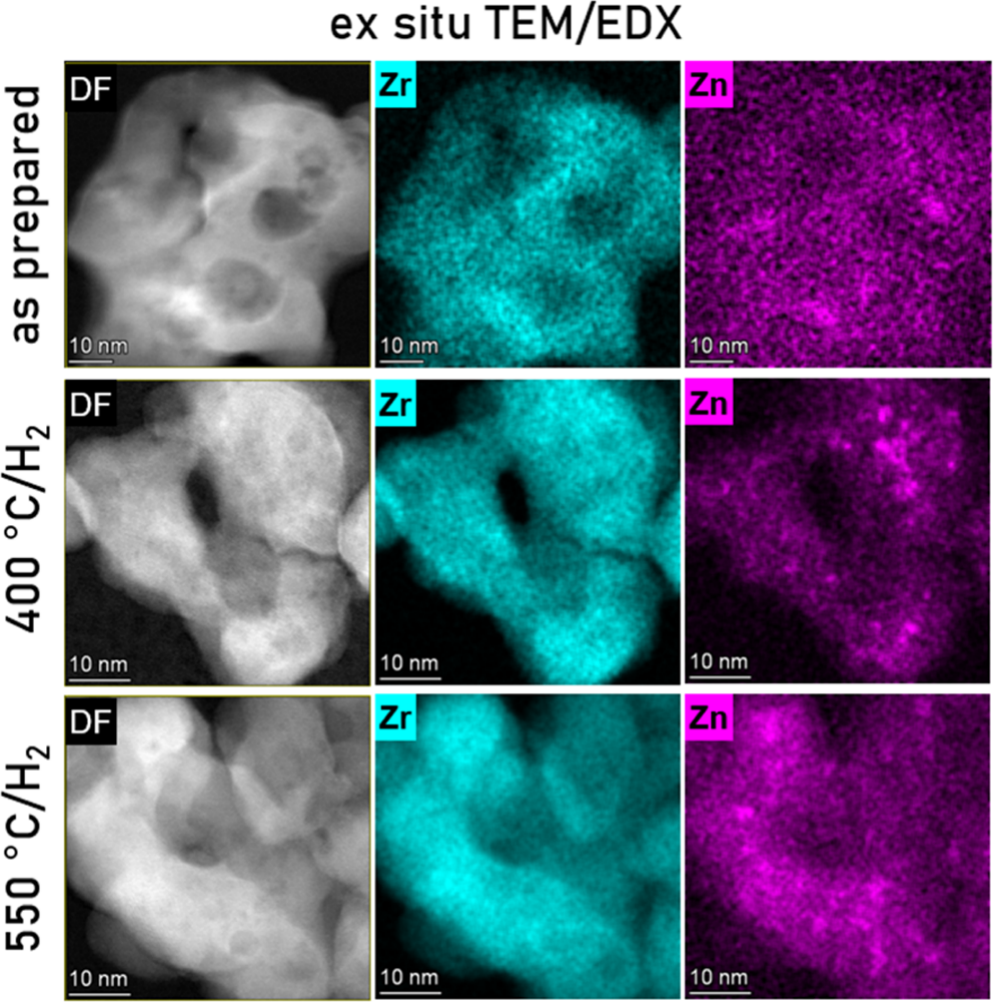
*Ex situ* STEM dark-field images (left column) and the EDX mapping of the corresponding areas (Zr, blue, middle column; Zn, purple, right column) for ZnZrO_*x*_-10 sample (as-prepared, top row; after 400 °C in H_2_/He, middle row; and after 550 °C in H_2_/He, bottom row).

Electron microscopy confirms the rearrangement of ZnO domains upon H_2_ pretreatments. Low-magnification STEM images (see Figures S16–S18) reveal an overall morphology of individual fused polycrystalline granules with characteristic nanoscale disordered pores/cavities. Due to the thickness and polycrystalline nature of the nanosized ZnO–ZrO_2_ phases, projection effects impeded the accuracy of the local structural analysis at high spatial resolution. TEM is generally consistent with a predominant t-ZrO_2_ phase in the ZnZrO_*x*_ catalysts. The decline in the volume of pores/cavities and lower surface area are apparent upon heat treatments and appear to be driven by the agglomeration of adjacent crystallites. Local EDX mapping of Zn and Zr is shown along with the corresponding dark-field STEM images in Figure 7 for the ZnZrO_*x*_-10 sample (top row, parent ZnZrO_*x*_-10; middle row, H_2_/He treated at 400 °C ZnZrO_*x*_-10–400; bottom row, H_2_/He treated at 550 °C ZnZrO_*x*_-10–550). Zn appears to be distributed homogeneously (at this magnification level) in some domains, which is consistent with solid solution, but also, multiple Zn-enriched areas of 3–10 nm size are apparent. Notably, the location of these Zn-rich nanoclusters is not correlated with the morphological features of the sample, *e.g.*, surface of the pores/cavities. Upon heating in H_2_/He at 400 °C, Zn clusters became more sharply defined and grew in numbers (see Figure S19), consistently with the early onset of Zn clustering suggested by *in situ* PXRD in Figure 6c. After the 550 °C treatment, the total amount of Zn predictably declined, and the contrast between the areas with homogeneous Zn distribution and Zn clusters is less pronounced with respect to the background (see Figure S20). However, clustered species clearly remained on the surface as the residual Zn species.

### Impact on Catalytic Performance

Methanol STY values ranging from 2.0 to 12.5 mol_methanol_ kg^–1^ h^–1^ were achieved under the range of operating conditions investigated in this work. In the literature, values up to 16 mol_methanol_ kg^–1^ h^–1^ can be found for these ZnZrO*_x_* catalysts;^2^ values ranging from 0.9 to 16 mol_methanol_ kg^–1^ h^–1^ were reported by Liu and Liu^32^ in their review on CuZn-based catalysts and similar values up to 10 mol_methanol_ kg^–1^ h^–1^ were reported for In_2_O_3_/ZrO_2_ catalysts.^33^ However, a direct comparison of measurements reported herein to these literature values is difficult because of the different conditions tested in each case, most of the time with significantly higher pressure than that used here. The main objective was to compare the performances of the catalysts after Zn was partially evaporated.

**Figure 8:**
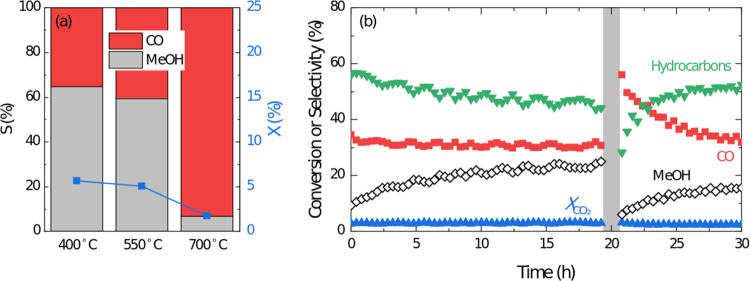
(a) CO_2_ conversion and methanol and CO selectivities over the ZnZrO_*x*_-10 sample as a function of prereduction temperature (reaction conditions, 350 °C, 30 bar, 12,000 cm^3^ h^–1^ g_ZnZrOx_^–1^, H_2_/CO_2_ ratio of 3) and (b) time on stream evolution of CO_2_ conversion and product selectivity over the ZnZrO_*x*_-10 + SAPO-18 (physically mixed in a 1:1 ratio) during a full reaction–regeneration-reaction cycle (reaction conditions, 350 °C, 30 bar, 48,000 cm^3^ h^–1^ g_ZnZrO*_x_*_^–1^, H_2_/CO_2_ ratio of 3).

The impact of Zn loss during H_2_ pretreatments at different temperatures on the kinetics of catalytic CO_2_ hydrogenation at standard reaction conditions (350 °C, 30 bar, GHSV 12,000–48,000 cm^3^ h^–1^ g_ZnZrO*_x_*_^–1^) is evaluated in Figure 8 for the ZnZrO_*x*_-10 catalyst. For all investigated space velocities, the conversion of CO_2_ remained well below the equilibrium line (see Figure S21), confirming that the conversion-selectivity data reflect the reaction kinetics. Methanol and CO were the only detected products. After the standard 400 °C pretreatment, CO_2_ conversion reached 6% with methanol selectivity slightly above 60%, which agrees well with our previous reports.^19^ Pretreatment at 550 °C did not significantly modify the CO_2_ hydrogenation performance, resulting in only a minor decrease in conversion and selectivity in comparison with the 400 °C pretreatment. The same result was confirmed for the 20 atom % sample (see Figure S22). Considering an appreciable Zn loss at this temperature (Figure 3a), the relatively unaltered catalytic performance after the 550 °C pretreatment suggests that not all evaporated Zn species participate in the formation of active sites. In fact, the estimated values of STY (in mol of methanol produced per mol of Zn and unit of time) suggest an increased rate of methanol formation after Zn loss: 0.12 and 0.14 min^–1^ after 400 and 550 °C reductive pretreatments, respectively. For example, a certain amount of Zn loss from the surface could be compensated by Zn migration out from the bulk. The unaltered Zr–Zn CN evaluated through a Zr K-edge EXAFS fit (Figure S10 and Table S2) also suggests limited variations in the ZnO–ZrO_2_ interface despite the loss of Zn, supporting these hypotheses. A further increase in the pretreatment temperature up to 700 °C led to a significant decrease in methanol selectivity to <10% and a conversion drop to <2%, which reflect more considerable loss of Zn from the material (STY, 0.02 min^–1^) and the absence of the Zr–Zn interface observed by FT-EXAFS fit (Figure S10 and Table S2). Methanol selectivity decrease suggests that the methanol- and CO-forming Zn species exhibit an unequal rate of volatilization and/or that the catalyst surface undergoes significant restructuring, leading to Zn redistribution in addition to evaporation. STY values for CO formation increase from 0.06 min^–1^ after 400 °C pretreatment and 0.09 min^–1^ after 550 °C pretreatment to 0.20 min^–1^ after 700 °C pretreatment.

Although pretreatment in H_2_/He up to 400 °C and in O_2_ up to 550 °C does not lead to Zn loss, characterization results presented earlier unequivocally show that Zn redistribution and material restructuring do occur at those conditions, including zinc migration, cluster growth, and ZrO_2_ phase transitions. In the final experiment, conditions that ZnZrO_*x*_ catalysts would experience in a technologically relevant CO_2_-to-hydrocarbon process were simulated over an entire pretreatment/reaction/regeneration/reaction cycle. Namely, ZnZrO_*x*_-10 sample was physically mixed with the methanol-to-hydrocarbon zeotype SAPO-18 catalyst synthesized in-house, which proved to be active for MTH at conditions of CO_2_ hydrogenation (see details in Xie et al.^34^). The catalyst mixture, with a 1:1 mass ratio, was pretreated in a H_2_/He flow at 400 °C, after which the flow was switched to a H_2_/CO_2_ (3:1) mixture, the temperature lowered to 350 °C, and the pressure increased to 30 bar. After almost 20 h on stream, during which a mixture of CO and hydrocarbons was produced by the tandem catalyst with 50% selectivity to hydrocarbons, the catalyst was regenerated by treatment in synthetic air at 550 °C (to remove the potentially formed coke from the zeotype channels), rereduced in a H_2_/Ar flow at 400 °C, and subjected to the same reaction conditions again. While the CO_2_ conversion remained the same after regeneration, hydrocarbon selectivity exhibited significant transient evolution (Figure 8b). After regeneration, selectivity declined to 30% with concomitant increase in CO production. As hydrocarbons are produced over the acid sites from methanol formed on the ZnO–ZrO_2_ interface, a modification in the mixed oxide morphology is suggested by this result. It took 5 h to regain the steady-state selectivity. It could be suggested that Zn mobility within the material is strongly influenced by the reduction/oxidation cycling, but *ex situ* analysis of the catalyst samples taken from the different stages of this experiment did not show significant variations in the Zn content at the surface. Taken into account that conventional XPS sampling depth encompasses many atomic layers (up to 3 nm at this incident energy), it is possible that near-surface restructuring is behind this selectivity changes without being visible in conventional XPS. We propose that oxidative treatment at 550 °C induces the growth of Zn clusters in the near-surface region of the catalyst, which would explain the increased CO production. The subsequent exposure to a partially reductive reaction environment gradually recovers the ZnO–ZrO_2_ interface selective to methanol *via* cluster breakup. A long-term stability test (see Figure S23) demonstrated that extended exposure to the standard reaction conditions (350 °C) does not affect either CO_2_ conversion or methanol selectivity, based on which the catalyst can be classified as thermally stable. However, stability should not be taken for granted at higher temperatures, which could otherwise be considered in future process optimization studies.

### Reconciled View of Zn Restructuring and Volatilization

**Figure 9:**
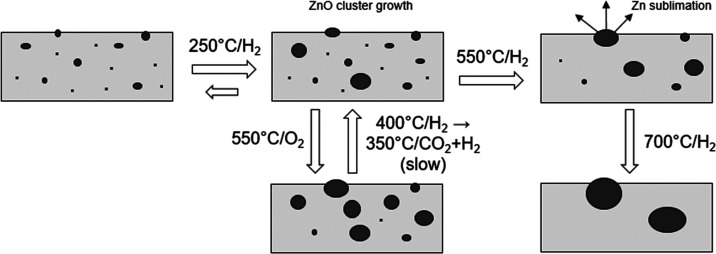
Environment-dependent ZnO cluster growth/redispersion and Zn loss by sublimation in the ZnZrO_*x*_ catalysts.

To summarize, based on the collection of data from multiple techniques discussed above, ZnZrO_*x*_ mixed oxide system undergoes structural transformations upon reductive pretreatments before and likely during the CO_2_ hydrogenation reaction. Initially, Zn is mostly present as small ZnO clusters embedded in the ZnO_2_ matrix, with possible minor contributions from close-to-atomically dispersed Zn ions (*i.e.*, solid solution), as suggested by *ex situ* TEM. Even relatively mild heating to 250–280 °C in the presence of hydrogen induces the migration of Zn, which results in ZnO cluster growth. Further heating up to 400 °C leads to decomposition of surface carbonates and partial reduction of the surface (leading to the formation of active sites for the catalytic reaction). However, the extent of these changes is limited, and they do not lead to Zn loss or selectivity decline. At even higher temperatures, ZnO cluster growth continues, but it is also accompanied by rapid evaporation of yet unknown, presumably monatomic Zn species into the gas phase (*i.e.*, sublimation). Zn egression from the ZrO_2_ matrix increases the unit cell size and destabilizes the tetragonal polymorph. We can conclude that the thermal stability of ZnZrO_*x*_ catalysts is strongly affected by the catalyst not being an ideal solid solution but rather being a nanoscale mixture of ZnO embedded and chemically bonded in the tetragonal ZrO_2_ matrix. As sketched in Figure 9, it can be hypothesized that at high temperature, ZnO cluster dimensions increase, facilitating Zn sublimation from ZnO nanodomains. This view is indirectly supported by the noticeably different rates of Zn loss from the two samples with different initial Zn concentrations, as shown in Figure 3a. The coordination environment of Zn changes for concentrations higher than 15%, as seen in the present study and reported in the literature before [*e.g.*, refs (2,16,19)], which can be assigned to the presence of bigger ZnO agglomerations physically deposited on the ZrO_2_ surface at these high loadings. Evidently, Zn volatilizes faster from these larger species, resulting in the steeper initial (negative) slope for the ZnZrO*_x_*-20 sample observed in Figure 3a. After Zn concentrations fall below 7%, both samples show similar and slower losses of Zn upon further increasing the temperature. Globally, this affects catalyst activity by (i) reducing ZrO_2_ tetragonal polymorph stability, which is more active than the monoclinic one and (ii) lowering the ZnO–ZrO_2_ interface where methanol-selective sites are actually located. With the decrease of the temperature, nanosized ZnO clusters are partially recovered, however, with a substantial reduction in the total Zn concentration and larger cluster size with respect to the as-prepared catalysts.

The nature and fate of the gas-phase Zn species are particularly intriguing. Sublimation experiments (see Figures 2 and 3) in which Zn was transferred *via* the gas phase revealed that at high-temperature conditions, only the ZrO_2_ surface, but not the quartz (SiO_2_) surface, is able to readsorb Zn. Interestingly, the deposited Zn imparts an intensely black color to the originally white ZrO_2_ (see Figure S1). The color can be attributed to ZnO nanostructures deposited from the gas phase, since XPS analysis (see Figure S4) did not reveal any carbon contamination above what was expected from the pure ZrO_2_ surface and both Zn 2p and Zn Auger spectra did not show any sign of metallic Zn. We propose that strong absorption in the visible range can be explained by the formation of in-gap states in partially reduced ZnO, which was previously reported in the literature.^35,36^ It is interesting to note the sharply contrasting bright white color of ZnO deposits downstream from the reactor (see Figure S1), where the temperature of the substrate (*i.e.*, quartz tube walls) was much lower. Moreover, no signs of metallic Zn(0) species were detected (by XPS) in either high- or low-temperature zones in the oxygen-free H_2_/He flow. The thickness of ZnO deposits on the tube walls excludes the possibility that the gas-phase Zn(0) species were reoxidized into Zn^2+^ by the terminal oxygen atoms on the quartz surface by mass-balance considerations. These observations motivate further research and bring into question conventionally proposed mechanisms of ZnO sublimation.

## Conclusions

Zn redistribution and volatility were investigated by several *ex situ* and *in situ* techniques in ZnZrO_*x*_ catalysts for CO_2_ hydrogenation into methanol, as a function of temperature and gas composition. The following conclusions can be drawn

- Zn^2+^ species are present in various forms in as-prepared materials, including ZnO clusters of 1–10 nm dimensions embedded in the ZrO_2_ matrix with minor contribution from atomically dispersed Zn ions in ZrO_2_.
- Upon heating in H_2_, Zn mobility manifests already at 250–280 °C. More specifically, ZnO clusters grow in size and the unit cell of the tetragonal phase becomes larger.
- Further heating induces destabilization of the tetragonal phase, and the cluster growth continues. Zn rapidly evaporates into the gas phase and is lost from the catalyst.
- Gaseous Zn species (no sign of metallic Zn(0) was found) can form ZnO deposits on pure ZrO_2_ at high temperature, which are highly defective and contain in-gap electronic levels related to oxygen vacancies, explaining intense light absorption in the visible range (“black ZnO”). However, minimal deposition on SiO_2_ surfaces at high temperature was detected (trace amounts, white color). At low temperature (*e.g.*, cold zone of the oven), these volatile Zn species form extended white ZnO deposits.
- Although Zn remains nonvolatile during high-temperature treatments in oxygen, such treatments lead to an unidentified surface restructuring that decreases methanol selectivity toward CO formation. Steady-state selectivity recovers within the following 5 h on stream.
- Observations of the ZnO cluster breakup upon cooling in PXRD experiments and selectivity recovery after oxidative treatments can possibly be explained by the formation of nonselective ZnO clusters at 550 °C in O_2_, which are gradually decreased in size and form more selective ZnO–ZrO_2_ interfaces upon treating the material in a reductive atmosphere at 400 °C in H_2_ and then restoring the reaction environment at 350 °C in a CO_2_/H_2_ mixture.

These findings offer novel insights into the formation, functioning, and eventual degradation of mixed oxide ZnZrO_*x*_ materials, which we hope will assist in the development of commercially viable tandem CO_2_ hydrogenation catalysts.

## Supporting Information Available

Quantification of the reaction performance and supporting characterization data (PXRD, DRIFTS, SEM/EDX, XAS, TEM) (PDF)
