# Supplementary material for: Zn Redistribution and Volatility in ZnZrOx Catalysts for CO2 Hydrogenation
Source: Chem Mater. 2023 Dec 11;35(24):10434–45. doi: 10.1021/acs.chemmater.3c01632 (PMC10753788; doi:10.1021/acs.chemmater.3c01632)
Supplement: Supplementary file 1 — cm3c01632_si_001.pdf [file cm3c01632_si_001.pdf]

# Supporting information for

# Zn re-distribution and volatility in $ZnZrO_x$ catalysts for $CO_2$ hydrogenation

Evgeniy A. Redekop[1,†,*], Tomas Cordero-Lanzac[1,†], Davide Salusso[2,†,a], Anuj Pokle[3], Sigurd Oien-Odegaard[1], Martin Fleissner Sunding[4], Spyros Diplas[4], Chiara Negri[1b], Elisa Borfecchia[2], Silvia Bordiga[2], and Unni Olsbye[1]

[1] Centre for Materials Science and Nanotechnology (SMN), Department of Chemistry, University of Oslo, N-0315 Oslo, Norway

[2] Department of Chemistry, NIS Center and INSTM Reference Center, via P. Giuria 7, University of Turin, 10125 Turin, Italy

[3] Centre for Materials Science and Nanotechnology (SMN), Department of Physics, University of Oslo, N-0315 Oslo, Norway

[4] Materials Physics Oslo, SINTEF Industry, Forskningsveien 1, NO - 0373 Oslo, Norway

[†] - co-authors contributed equally to this work

[*] - corresponding author

[a] – current affiliation: European Synchrotron Radiation Facility, CS 40220, 38043 Grenoble Cedex 9, France

[b] – current affiliation: Department of Energy, Politecnico di Milano, Via Lambruschini, 4, 20156 Milano, Italy

**S1. Catalytic performance**

CO2 hydrogenation was monitored by the definition of CO2 conversion,

$$X_{\mathrm{CO_2}} = \frac{F_{\mathrm{CO}} + F_{\mathrm{OX}}}{F^{in}_{\mathrm{CO_2}}} 100 \quad , \tag{S1}$$

where FCO and FOX are the molar flow rate in contained C units of CO and oxygenates (OX) at the outlet of the reactor and FinCO2 is the molar flow rate of CO2 at the entrance of the reactor. The selectivity to CO and oxygenates (mainly methanol in our case) was defined as

$$S_i = \frac{F_i}{F_{\mathrm{CO}} + F_{\mathrm{OX}}} 100, \quad i = \mathrm{CO}, \mathrm{OX} \quad , \tag{S2}$$

where Fi is the molar flow rate in contained C units of either CO or oxygenates (OX) at the outlet of the reactor. Space time yields of methanol and CO (STY) has been defined as a function of the space time (τ)

$$\mathrm{STY}_i = \frac{X_{\mathrm{CO_2}} S_i}{\tau}, \quad i = \mathrm{CO}, \mathrm{OX} \quad , \tag{S3}$$

where the space time is define in units of mmolCO2 (fed) h gcatalyst-1 or molCO2 (fed) min molZn-1 (in the catalyst after the pretreatment).

## S2. Supplementary Figures

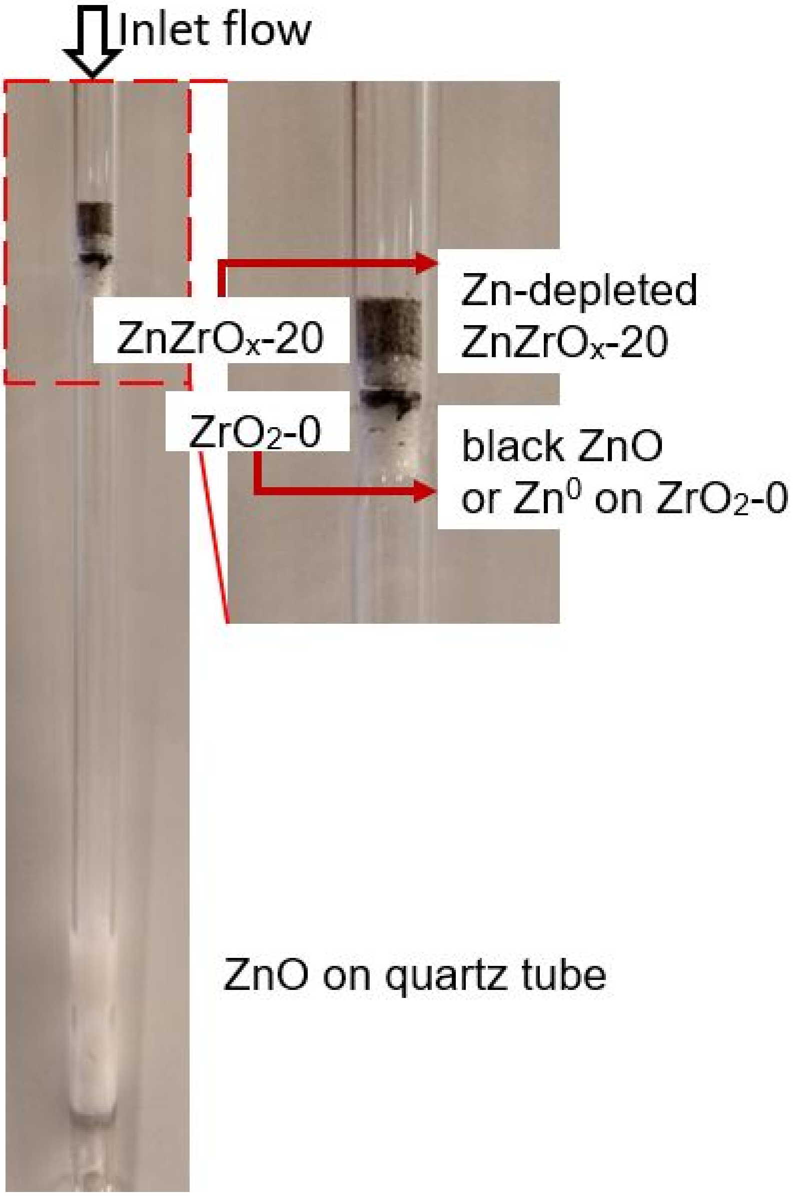


Figure S1. Apparatus for sublimation experiments

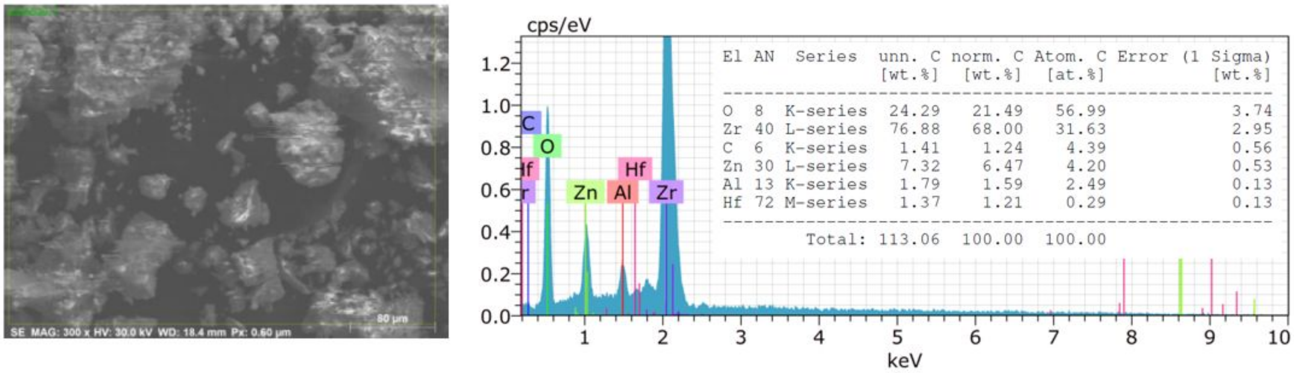


Figure S2. Example SEM/EDX quantification of Zn content, in this case, in $ZnZrO_x$-10 sample

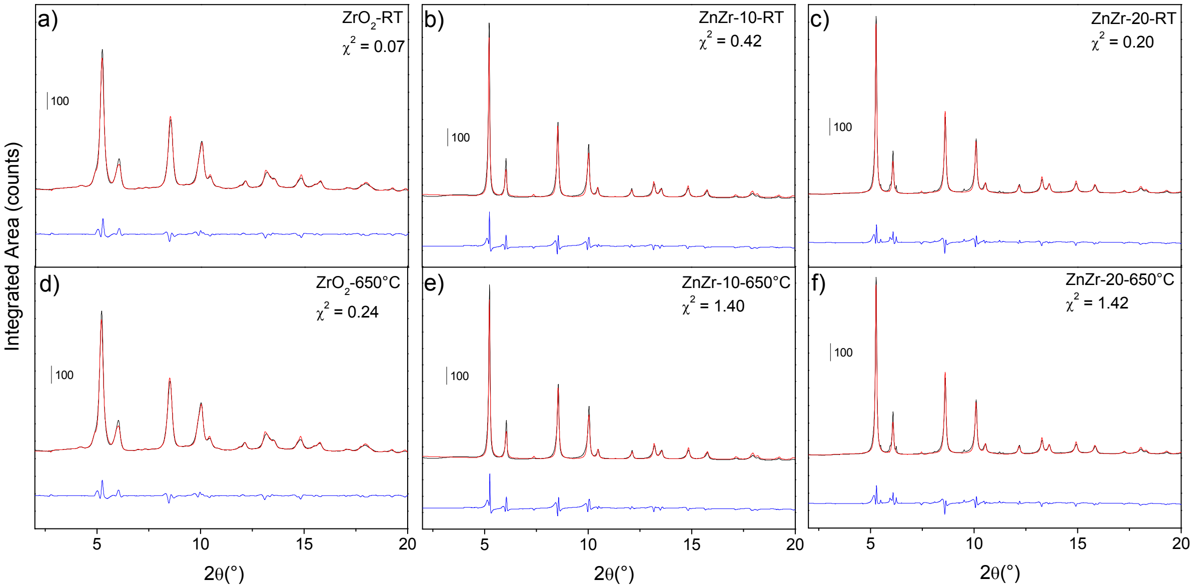


Figure S3. Experimental PXRD pattern (black line), refined curve (red line) and difference function for a,d) $ZrO_2$, b,e) $ZnZrO_x$-10 and c,f) $ZnZrO_x$-20 under $H_2$ at RT (a,b,c) and at 650 °C (d,e,f).

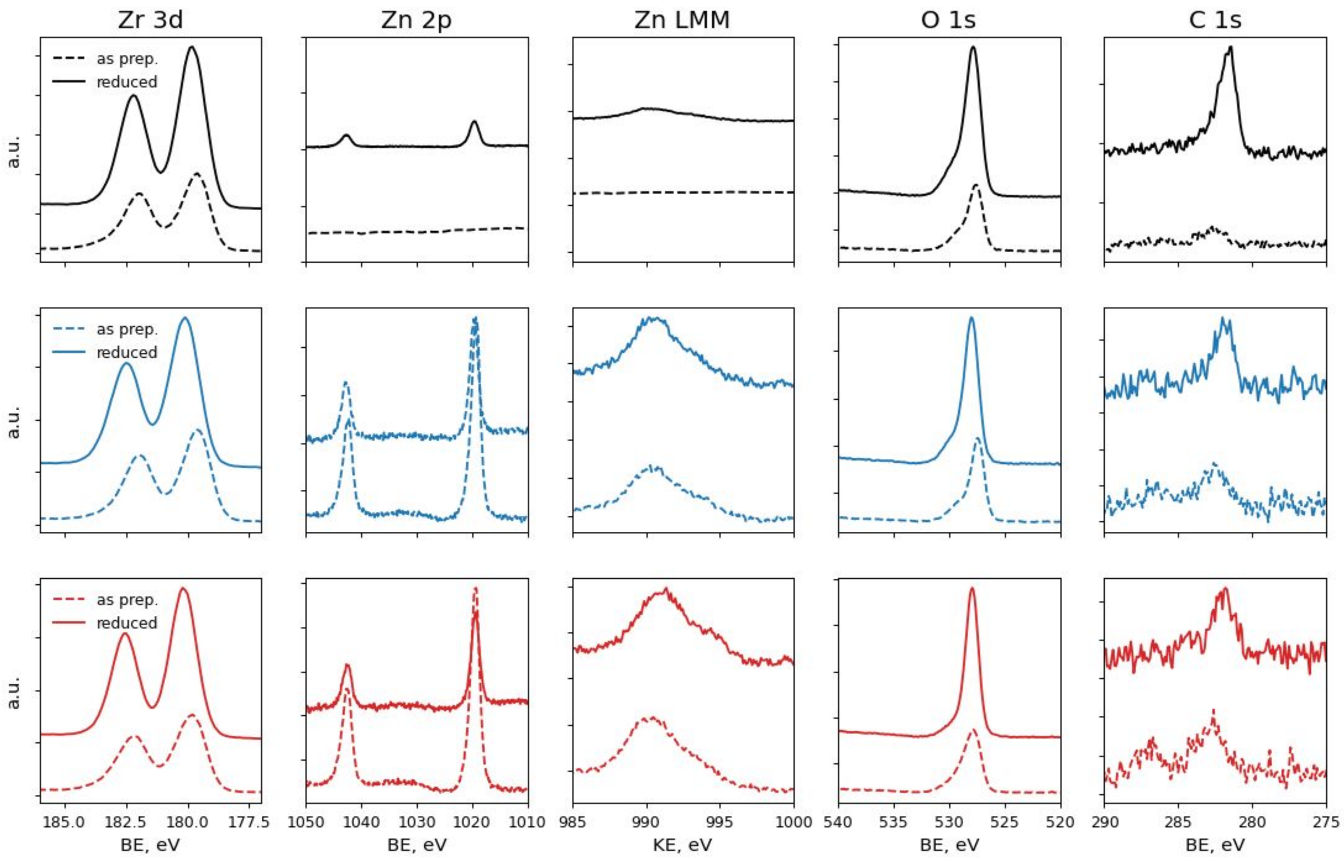


Figure S4. XPS spectra before (dashed) and after (solid) reduction in $H_2$: $ZnZrO_x$-0 (top), $ZnZrO_x$-10 (middle), and $ZnZrO_x$-20 (bottom).

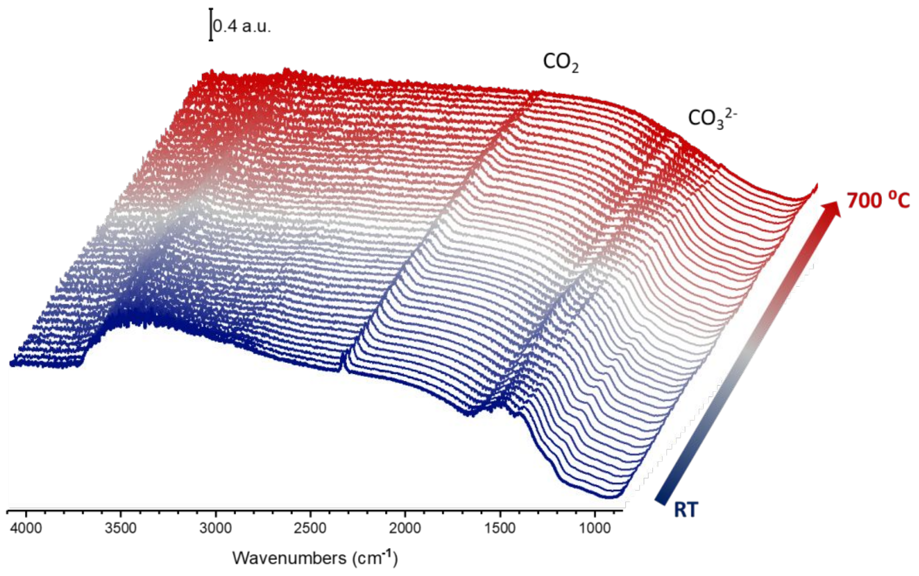


Figure S5. DRIFTS FTIR during $H_2$ TPR of $ZnZrO_x$-10 catalyst.

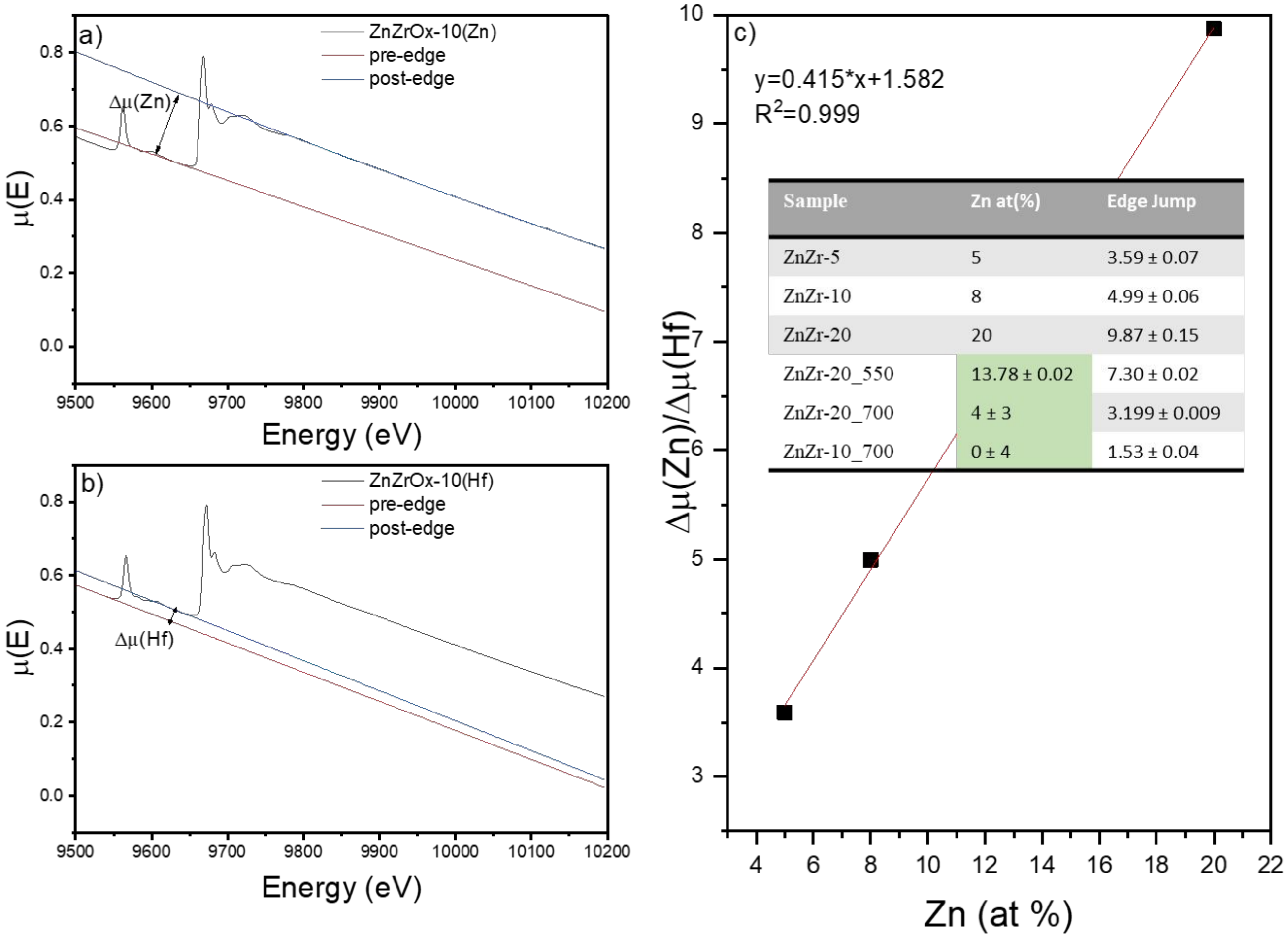


| Sample | Zn at(%) | Edge Jump |
|---|---|---|
| ZnZr-5 | 5 | 3.59 ± 0.07 |
| ZnZr-10 | 8 | 4.99 ± 0.06 |
| ZnZr-20 | 20 | 9.87 ± 0.15 |
| ZnZr-20_550 | 13.78 ± 0.02 | 7.30 ± 0.02 |
| ZnZr-20_700 | 4 ± 3 | 3.199 ± 0.009 |
| ZnZr-10_700 | 0 ± 4 | 1.53 ± 0.04 |

Figure S6. Zn K-edge raw absorption data of ZnZrOx-10 reporting pre-edge and post-edge lines employed for normalization at a) Zn K- and b) Hf $L_3$-edges. c) Calibration line obtained using ratio between Zn K- and Hf $L_3$-edge steps extracted as indicated in panels a,b. The table reports the employed Zn at. % and edge jump original data. Boxes in green indicate Zn at. % values extracted using the calibration line.

EXAFS fit at Zn K-edge was not conducted due to limited spectra quality. Indeed, while all the FT-EXAFS where extracted in the 2.5 – 10.4 Å$^{-1}$ k-range, the spectra of ZnZrO-10-700 was extracted until k 8 Å$^{-1}$ (Figure S7a). This reduced the number of available independent variables from 10 (2.5 – 10.4 Å$^{-1}$ k-range) to 7 (2.5 – 8 Å$^{-1}$ k-range). In both cases the spectra quality was not sufficient to reproduce the fit previously reported. Nevertheless, from a qualitative perspective we observed that ZnZrO-10-700 and ZnZrO-20-700 presented fingerprints of Zn-Zn scattering path from ZnO in both k space and $k^2$-weighted FT-EXAFS magnitude and imaginary parts (indicated with arrows in Figure S7), indicating an increase of the ZnO cluster dimension.

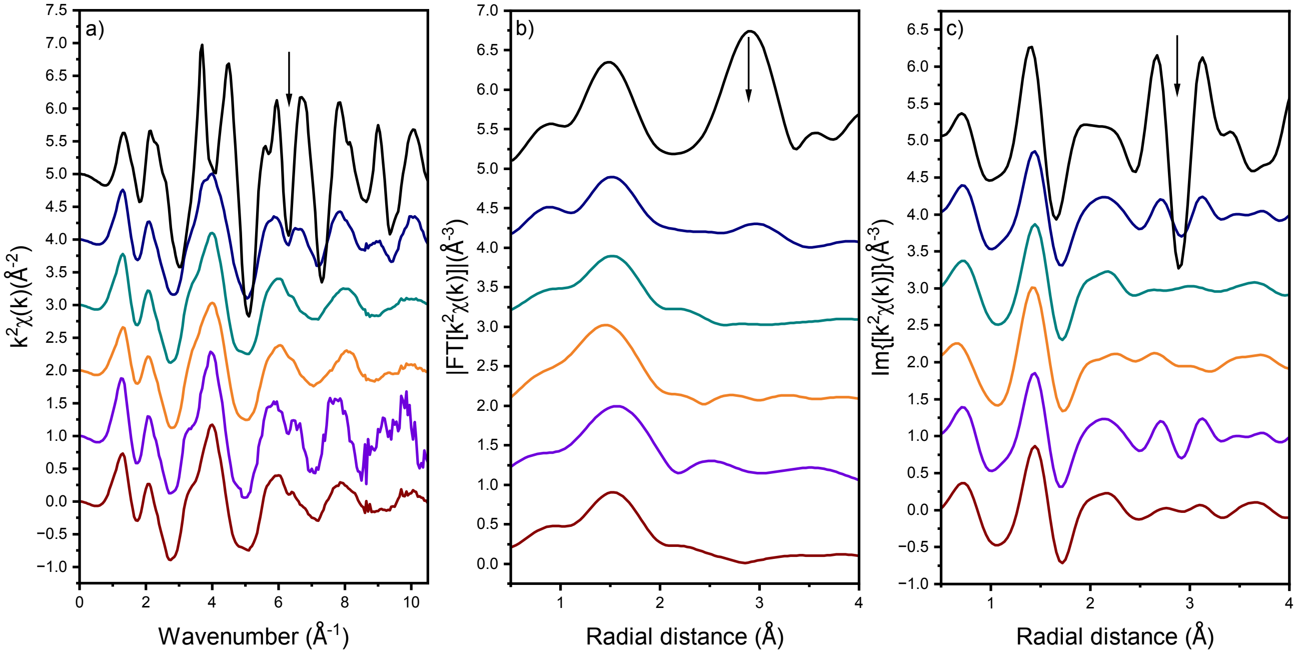


Figure S7 Zn K-edge $k^2$-weighted EXAFS (a) spectrum and its Fourier Transform magnitude (b) and imaginary (c) parts of (from bottom to top) ZnZrO-10 (dark red line), ZnZrO-10-700 (purple line), ZnZrO-20 (orange line), ZnZrO-20-550 (dark cyan line), ZnZrO-20-700 (blue line) and h-ZnO (black line).

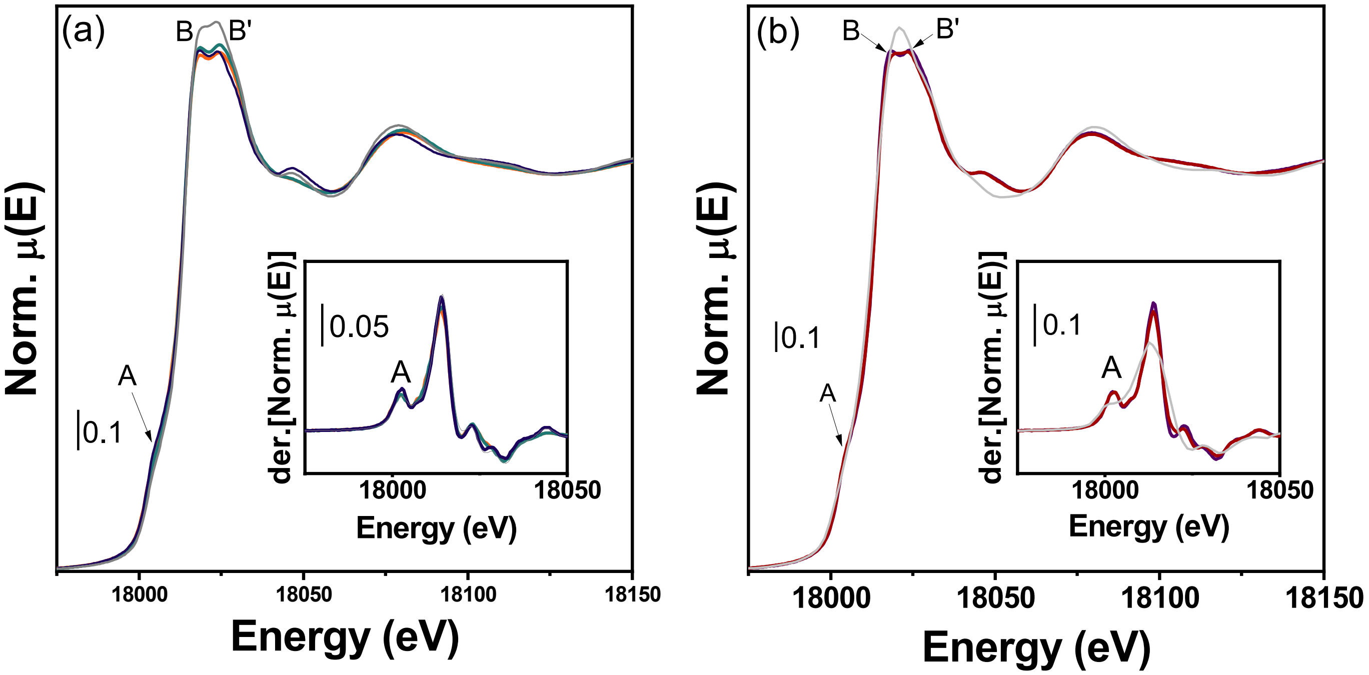


Figure S8. Zr K-edge XANES spectra of a) ZnZrO-10 (dark red line), ZnZrO-10-700 (purple line) and ZnZrOx-20 as prepared (orange line), ZnZrOx-20-550 (light blue line) and ZnZrOx-20- 700 (dark blue). t-ZrO2 reference spectrum is reported in grey. XANES first derivative spectra are reported in bottom insets.

EXAFS fit of spectra collected at Zr K-edge was conducted for all the samples. Zr-O, Zr-Zr and Zr-Zn single scattering path were calculated using FEFF6 considering the structural model previously reported presenting a Zr atom surrounded by a double tetrahedra configuration of oxygen atoms in first coordination shell and [(12-Zn)*Zr+Zn] atoms in second coordination shell [main text, ref 15]. The scattering amplitude factor was determined from fitting of Zr foil and fixed to the found value (see Figure S9 and Table S1). To estimate the content of Zn, the coordination numbers of Zr-Zr and Zr-Zn scattering paths were refined as 12-Zn and Zn, respectively, where the Zn variable describes the amount of Zn atoms surrounding the absorber Zr atom (Figure S10 and Table S2). However, to reduce the correlation with the scattering path Debye Waller factor contribution, the latter was fixed to the values determined with the Einstein model in our previous work [main text, ref 15]. As previously reported, this approach

can provide a rough estimation of the Zn content in the material. The best-fit values of Zn ≈ 0 obtained in the Zr K-edge EXAFS fits for ZnZrO-10, ZnZrO-10-700 and ZnZrO-20-700 suggest that the amount of Zn atoms located at the ZnO nano-cluster/ZrO2 matrix interface (and thus contributing to the Zr-Zn scattering path) in these cases is lower than the detection limit of the technique (≈15%, considering the error evaluated from the fit of ZnZrO-20).

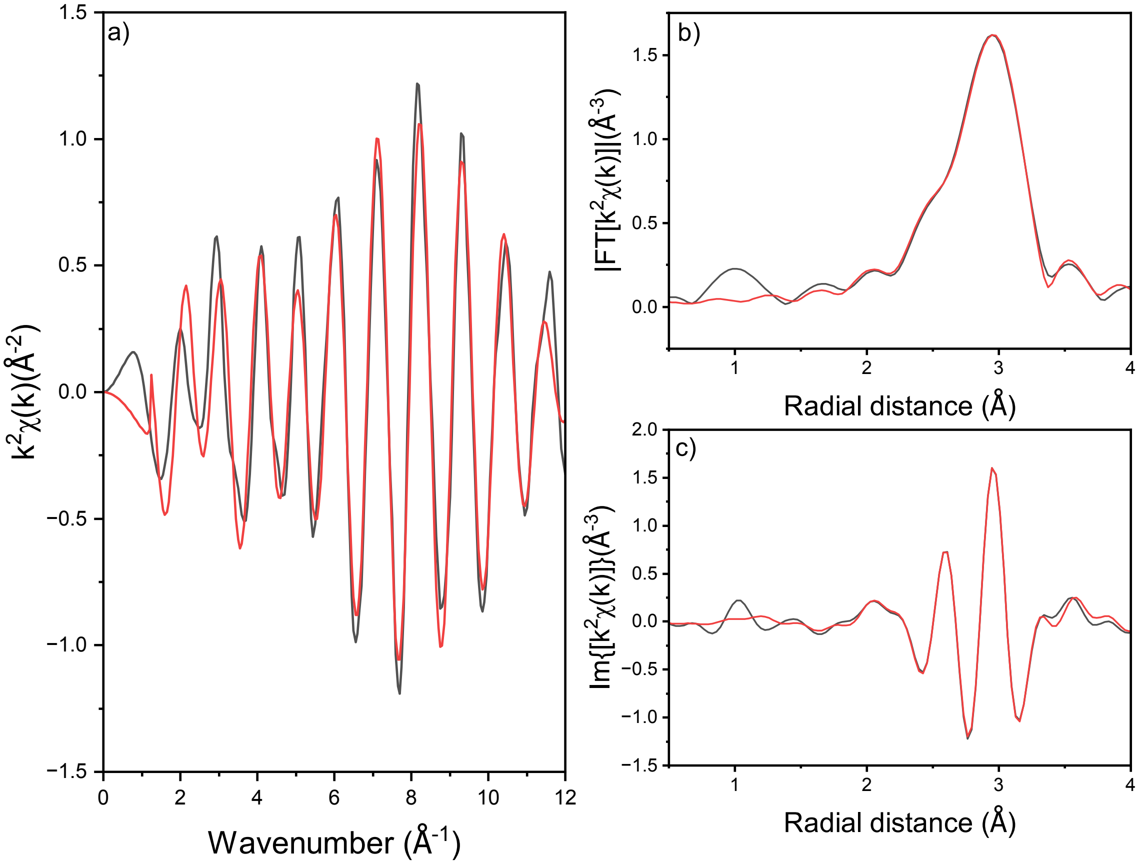


Figure S9 Experimental spectra (black line) and best fit (red line) of Zr metal Zr K-edge $k^2$-weighted EXAFS spectra (a) and its Fourier Transform magnitude (b) and imaginary (c) parts.

Table S1 EXAFS fit results of Zr K-edge FT-EXAFS of Zr metal. FT-EXAFS was extracted in the 3 – 11.5 $Å^{-1}$ k-range while fit was performed in the 1 – 3.3 $Å^{-1}$ R-range. Metallic Zr with space group P 63/m m c was used as input structure for FEFF6 to calculate the two employed Zr-Zr single scattering paths describing the spectra first coordination shell.

| | |
|---|---|
| R-factor | 0.0089 |
| $N_{var}(N_{ind})$ | 6(12) |
| $S_0^2$ (passive amplitude reduction factor) | $0.68 \pm 0.10$ |
| $\Delta E$ (eV) | $5.5 \pm 0.9$ |
| $\sigma^2_{Zr(I)}$(Å$^2$) | $0.001 \pm 0.002$ |
| $R_{Zr(I)}$(Å) | $3.133 \pm 0.011$ |
| $\sigma^2_{Zr(II)}$(Å$^2$) | $0.001 \pm 0.002$ |
| $R_{Zr(II)}$(Å) | $3.261 \pm 0.010$ |

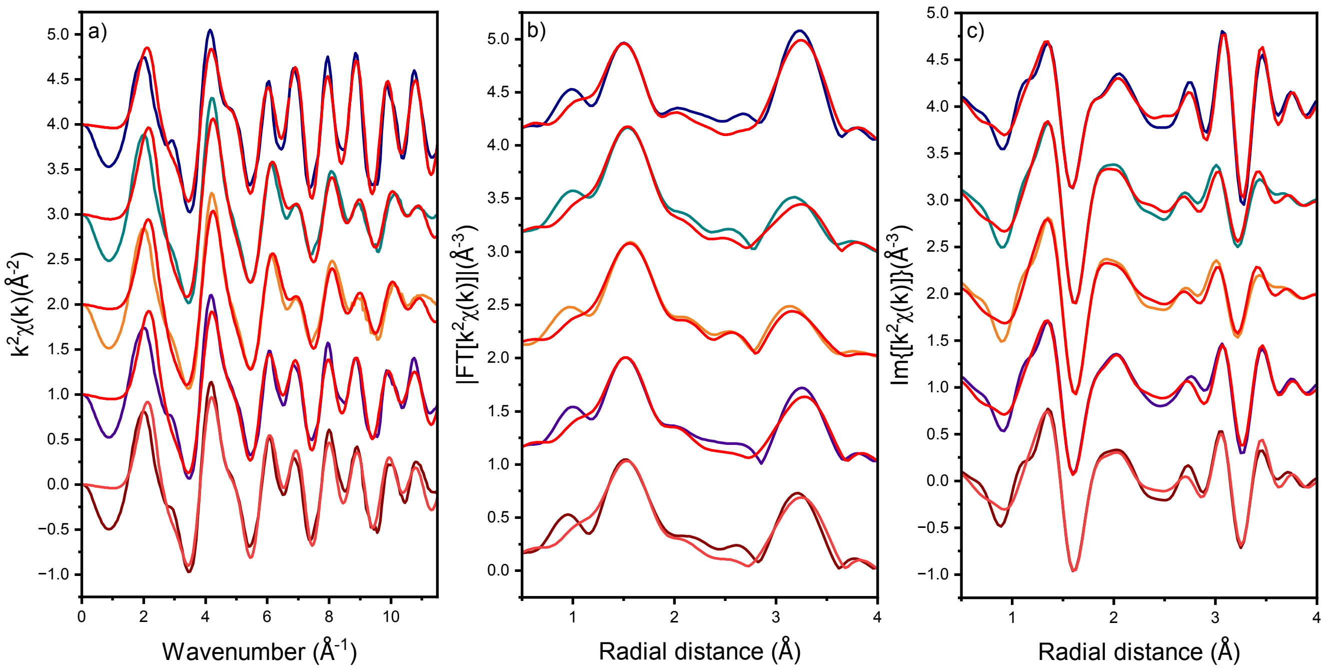


Figure S10 Experimental spectra and best fit (red lines) of Zr K-edge $k^2$-weighted EXAFS spectra (a) and their Fourier Transform magnitude (b) and imaginary (c) parts of ZnZrO-10 (dark red line), ZnZrO-10-700 (purple line), ZnZrO-20 (orange line), ZnZrO-20-550 (dark cyan line) and ZnZrO-20-700 (blue line).

Table S2 EXAFS fit results of Zr K-edge FT-EXAFS of the studied samples. FT-EXAFS were extracted in the 2.5-11.2 (Å$^{-1}$) k-range and fit performed in the 1 – 3.65 Å range. Passive amplitude reduction factor S02 fixed to 0.68 as determined from EXAFS fit of Zr metal reported above. * indicates parameters fixed to values previously evaluated. ** The variable 'Zn' indicates the number of Zn atoms replacing the 12 Zr atoms surrounding the absorber Zr in second coordination shell.

| | ZnZrO-10 | ZnZrO-10-700 | ZnZrO-20 | ZnZrO-20-550 | ZnZrO-20-700 |
|---|---|---|---|---|---|
| R factor | 0.03 | 0.019 | 0.03 | 0.03 | 0.03 |
| $N_{var}(N_{ind})$ | 8(14) | 8(14) | 8(14) | 8(14) | 7(14) |
| ΔE (eV) | -2.3 ± 1.8 | -1.4 ± 1.6 | -2 ± 2 | -2.1 ± 1.9 | -2.6 ± 1.5 |
| $\sigma^2_{O(I)}$(Å$^2$) | 0.003 ± 0.002 | 0.0023 ± 0.0016 | 0.003 ± 0.002 | 0.002 ±0.001 | 0.002 ± 0.001 |
| $R_{O(I)}$(Å) | 2.094 ± 0.019 | 2.093 ± 0.015 | 2.09 ± 0.02 | 2.089 ± 0.018 | 2.084 ± 0.014 |
| $\sigma^2_{O(II)}$(Å$^2$) | 0.008 ± 0.005 | 0.006 ± 0.003 | 0.005 ± 0.003 | 0.004 ± 0.003 | 0.010 ± 0.005 |
| $R_{O(II)}$(Å) | 2.25 ± 0.03 | 2.27 ± 0.03 | 2.24 ± 0.03 | 2.24 ± 0.03 | 2.27 ± 0.03 |
| $\sigma^2_{Zr}$(Å$^2$) | 0.0095* | 0.0095* | 0.0095* | 0.0095* | 0.0064 ± 0.0006 |
| $R_{Zr}$(Å) | 3.63 ± 0.02 | 3.64 ± 0.02 | 3.60 ± 0.06 | 3.60 ± 0.05 | 3.634 ± 0.010 |
| $\sigma^2_{Zn}$(Å$^2$) | 0.006 | 0.006* | 0.006* | 0.006* | / |
| $R_{Zn}$(Å) | 3.46 ± 0.08 | 3.703 ± 0.017 | 3.59 ± 0.14 | 3.60 ± 0.15 | / |
| Zn** | 0 ± 1 | 0 ± 2 | 2.2 ± 1.9 | 2 ± 2 | / |

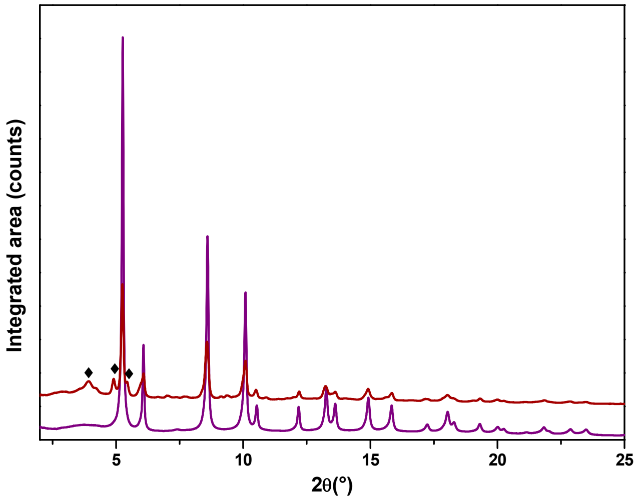


Figure S11. Stacked *ex situ* PXRD of ZrZnOx-10 (purple line) and ZrZnOx-10-700 (red line). Main monoclinic Bragg reflections are indicated with diamonds.

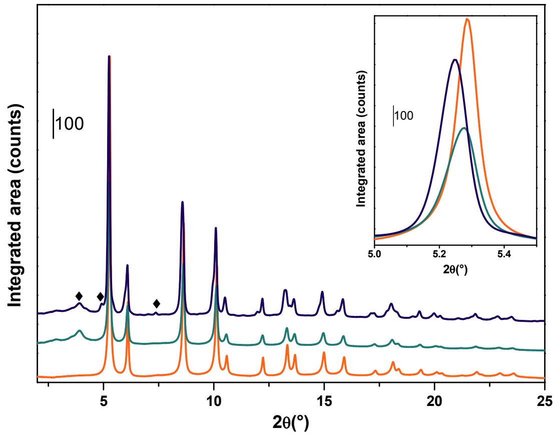


Figure S12. Stacked *ex situ* PXRD of ZnZnOx-20 (orange line), ZrZnOx-20-550 (green line) and ZrZnOx-20-700 (blue line). Main monoclinic Bragg reflections are indicated with diamonds. Detail of t-$ZrO_2$ (110) reflection is showed in the inset.

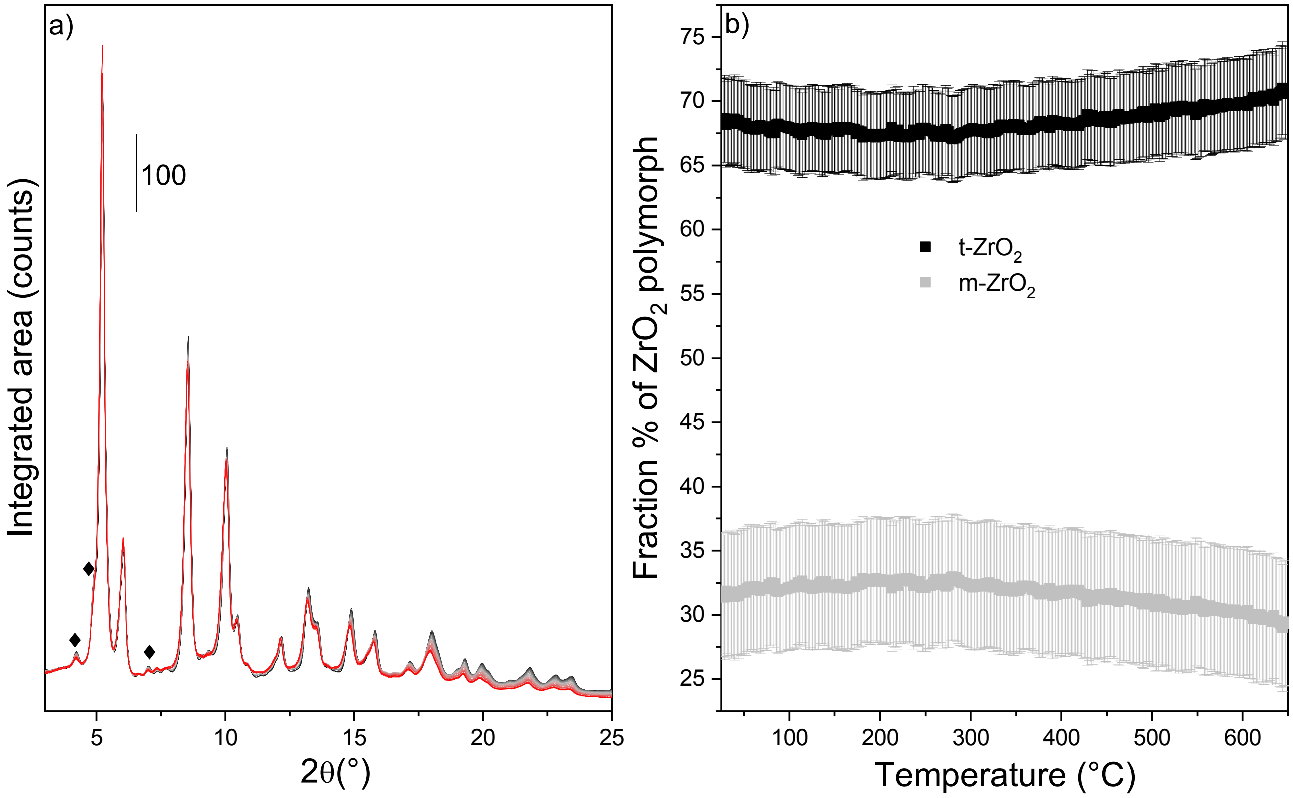


Figure S13. a) $ZrO_2$ *in situ* PXRD data collected during heating under $H_2$ flow. Temperature increases from black to red line. b) Fraction of monoclinic/tetragonal $ZrO_2$ calculated by Rietveld refinement.

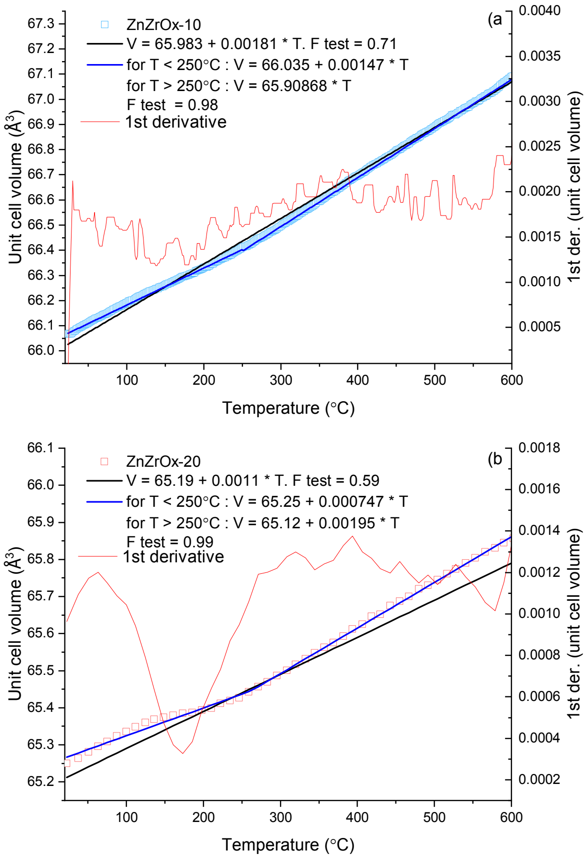


Figure S14. 1st derivative (red line) of unit cell volume variation with temperature (open squares) of a) $ZnZrO_x$-10 and b) $ZnZrO_x$-20. Linear fit using a single (black line) and two components (blue line) are shown. Linear fit equations and F test results are displayed in the legend.

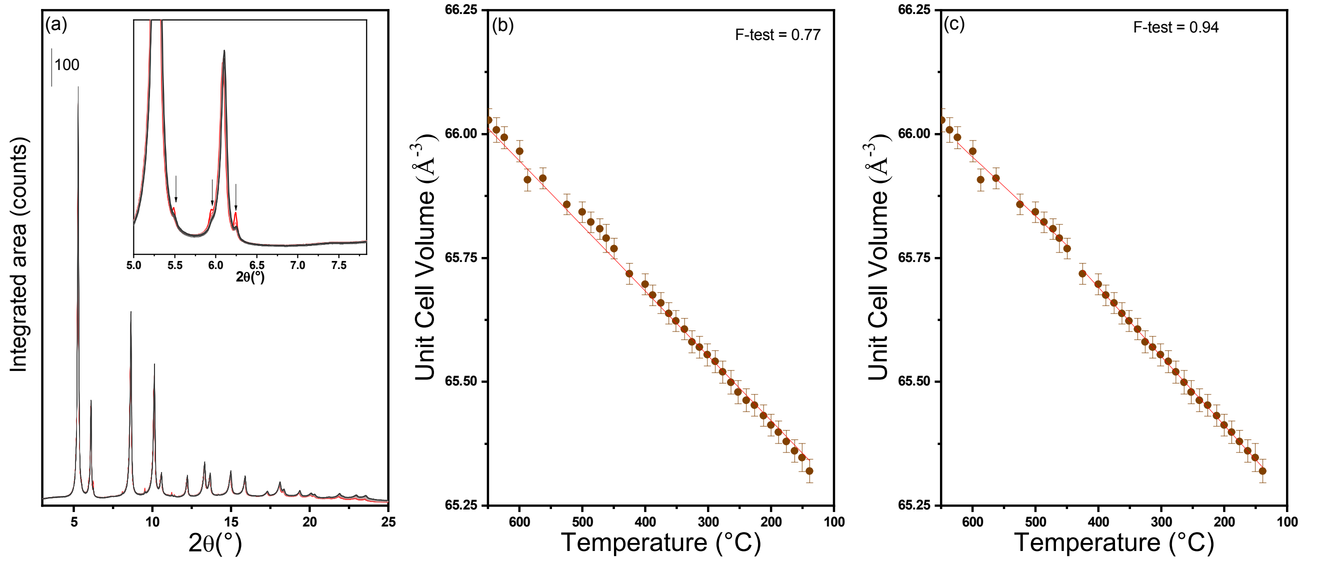


Figure S15. a) $ZnZrO_x$-20 *in situ* PXRD data collected after $H_2$-TPR experiment during cooling under $H_2$/He flow. Temperature decreases from red (650 °C) to black (RT) line. Detail of h-ZnO reflections (indicated with arrows) is showed in the inset. b,c) Linear fit of unit cell volume contraction during cooling considering b) a single region and c) two regions are shown. F-test of the two fit are reported on the respective panels.

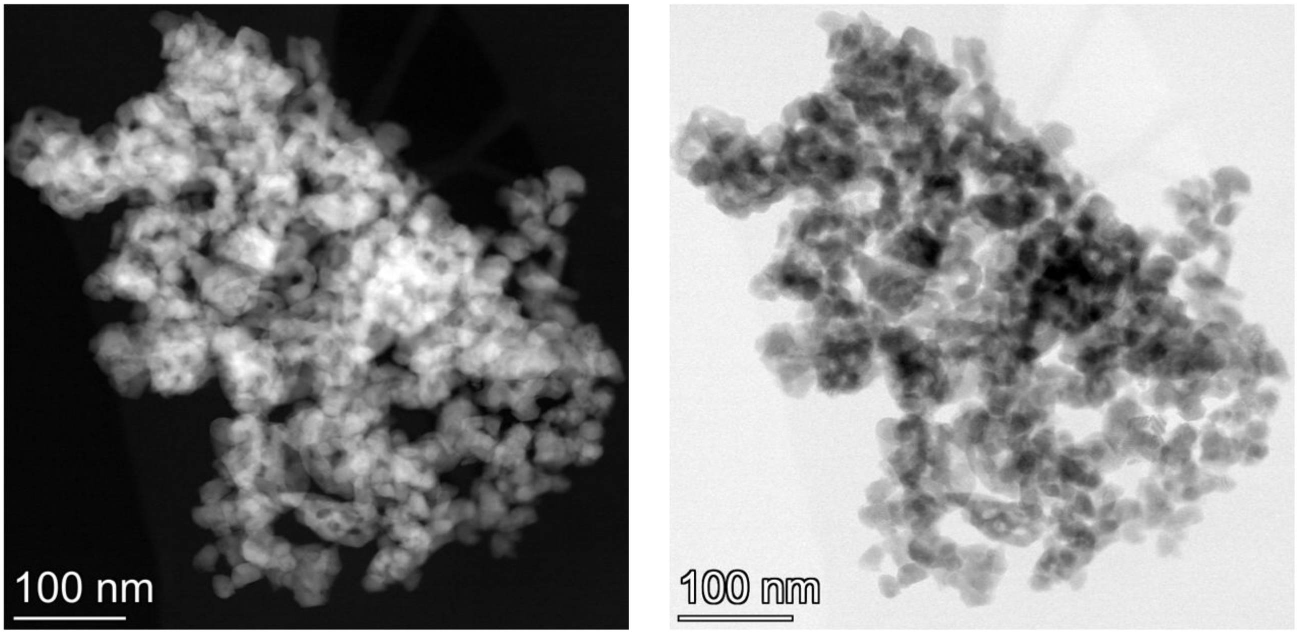


Figure S16. Dark (left) and bright (right) field images of as prepared $ZnZrO_x$-10 sample.

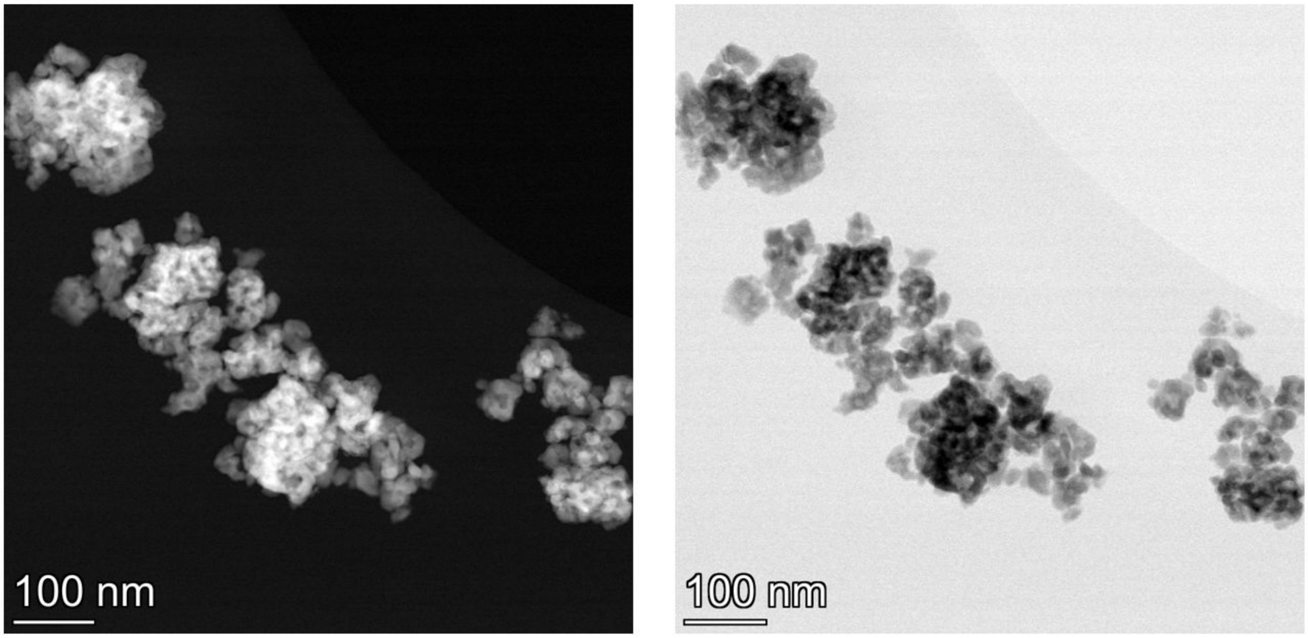


Figure S17. Dark (left) and bright (right) field images of $ZnZrO_x$-10 sample after 400 °C pretreatment in 50 % $H_2$ (in He) flow.

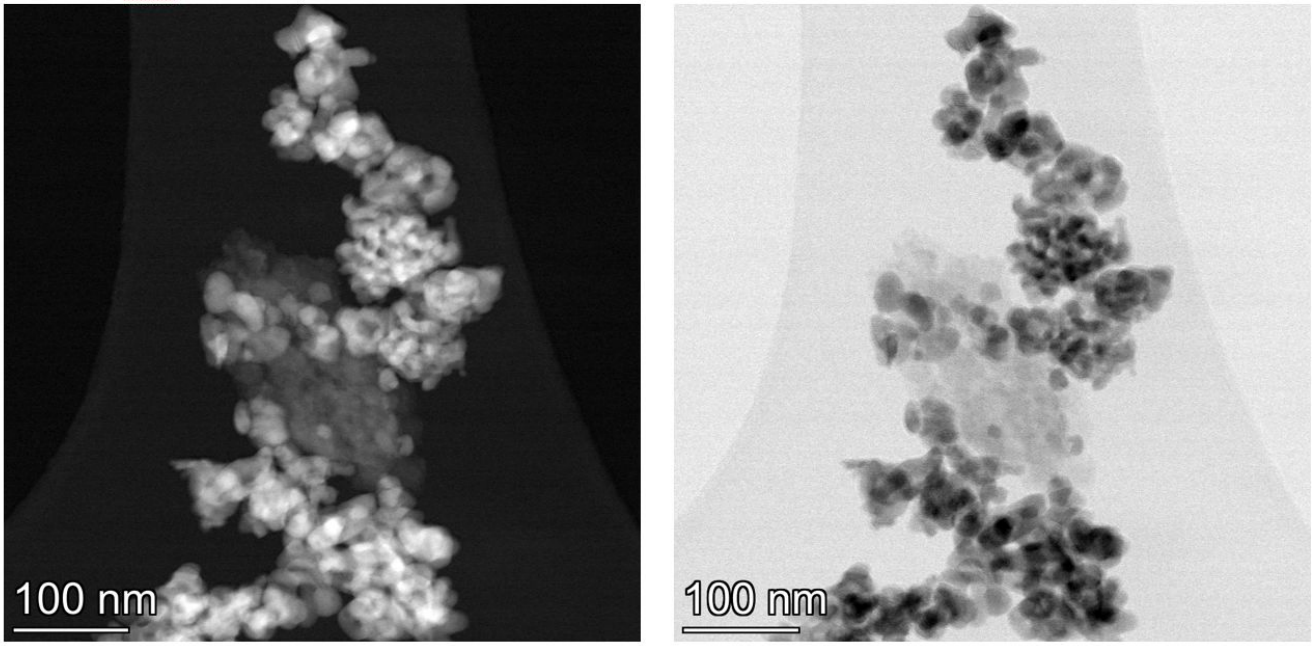


Figure S18. Dark (left) and bright (right) field images of $ZnZrO_x$-10 sample after 550 °C pretreatment in 50 % $H_2$ (in He) flow.

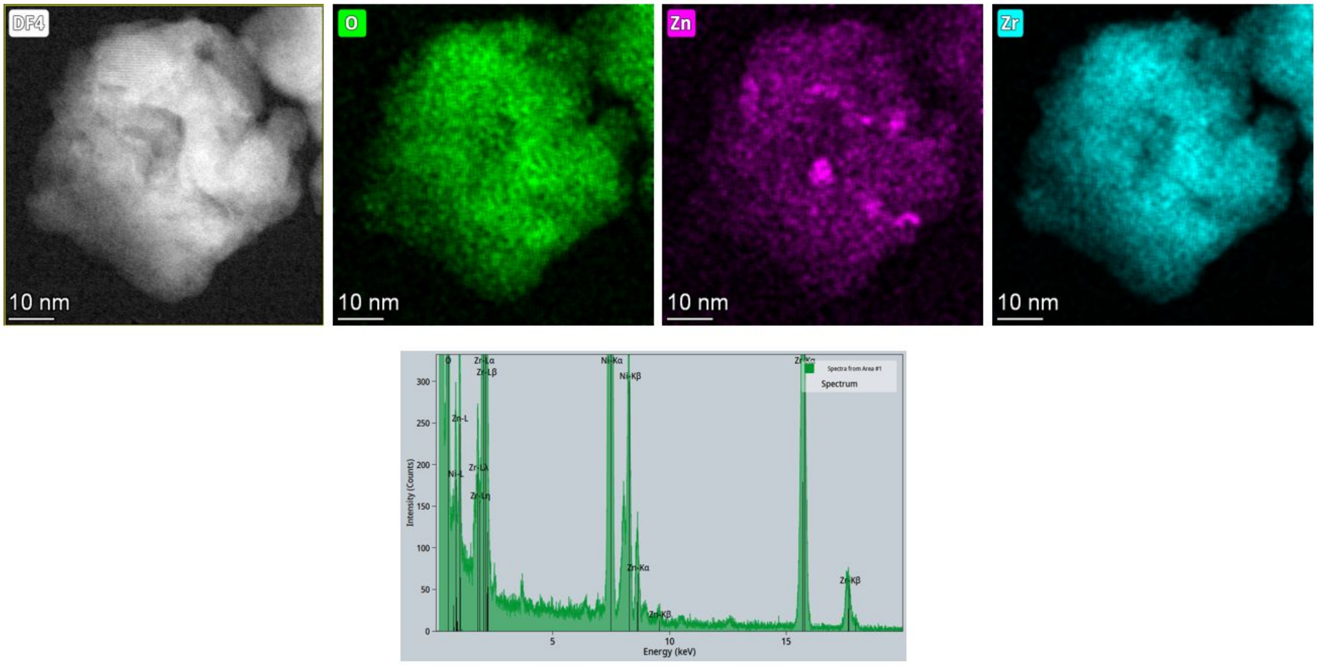


Figure S19. Dark image (left) and EDX maps of $ZnZrO_x$-10 sample after 440 °C pretreatment in 50 % $H_2$ (in He) flow.

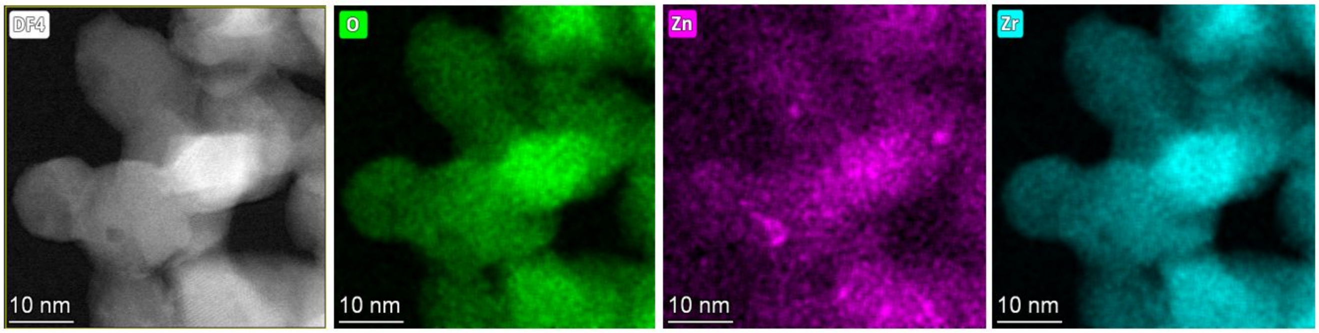


Figure S20. Dark image (left) and EDX maps of $ZnZrO_x$-10 sample after 550 °C pretreatment in 50 % $H_2$ (in He) flow.

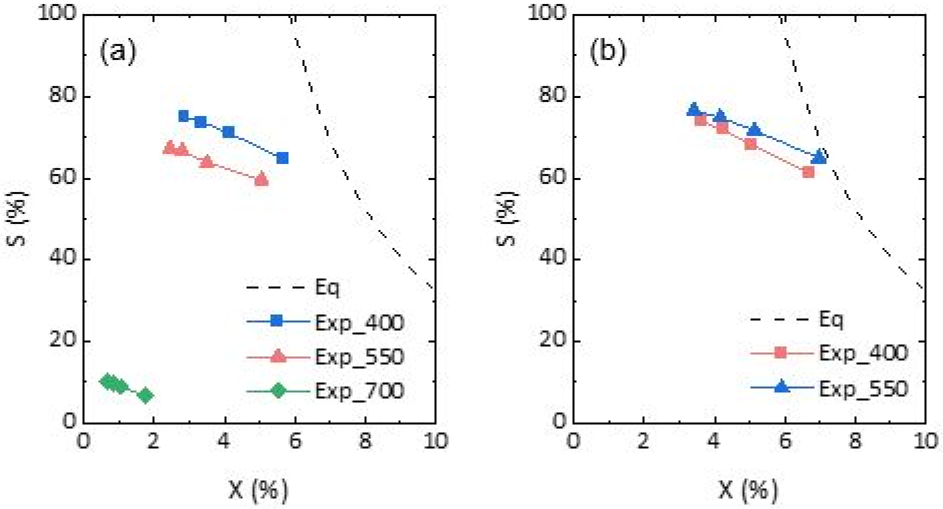


Figure S21. Evolution with $CO_2$ conversion of methanol selectivity for the (a) ZnZrOx-10 and (b) ZnZrOx-20 catalysts after pretratment at different temperatures (reaction conditions 350 ºC, 30 bar 12000-48000 $cm^3\ h^{-1}\ g_{ZnZrOx}^{-1}$). Dashed line is the $CO_2$-to-methanol equilibrium limitation.

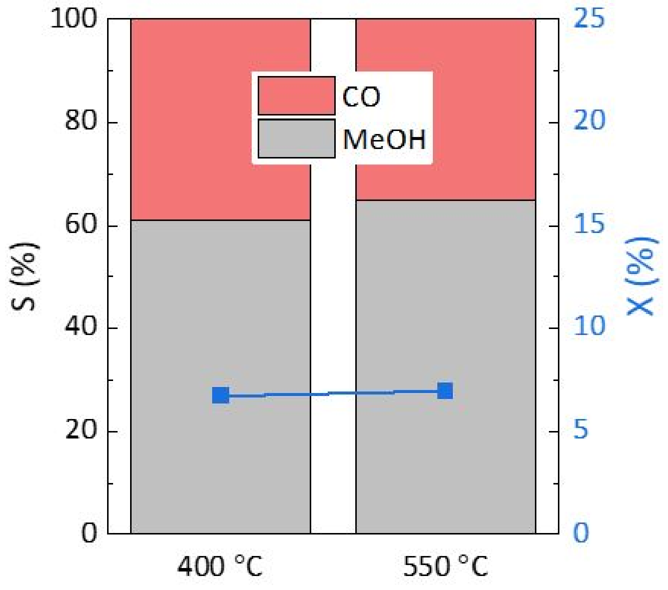


Figure S22. Impact of 400-550 ºC $H_2$/He pretreatment on the performance of $ZnZrO_x$-20 catalyst (reaction conditions 350 ºC, 30 bar 12000 $cm^3\ h^{-1}\ g_{ZnZrOx}^{-1}$).

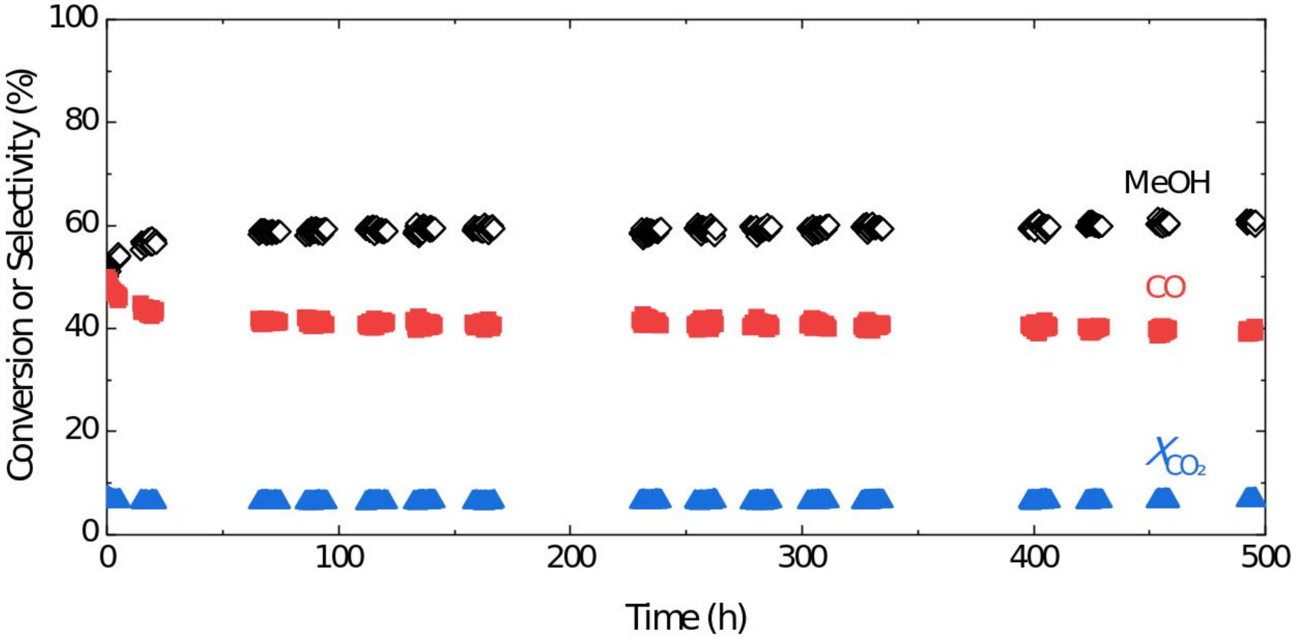


179 Figure S23. Impact of prolonged exposure of the ZnZrOx-15 catalyst to the standard reaction
180 conditions (350 ºC).
